# Supplementary material for: Mammalian antiviral proteins ZAP and KHNYN can independently restrict CpG-enriched avian viruses
Source: PLoS Biol. 2025 Oct 28;23(10):e3003471. doi: 10.1371/journal.pbio.3003471 (PMC12582503; doi:10.1371/journal.pbio.3003471)
Supplement: S1 Raw Images — (PDF) [file pbio.3003471.s020.pdf]

Figure 2E

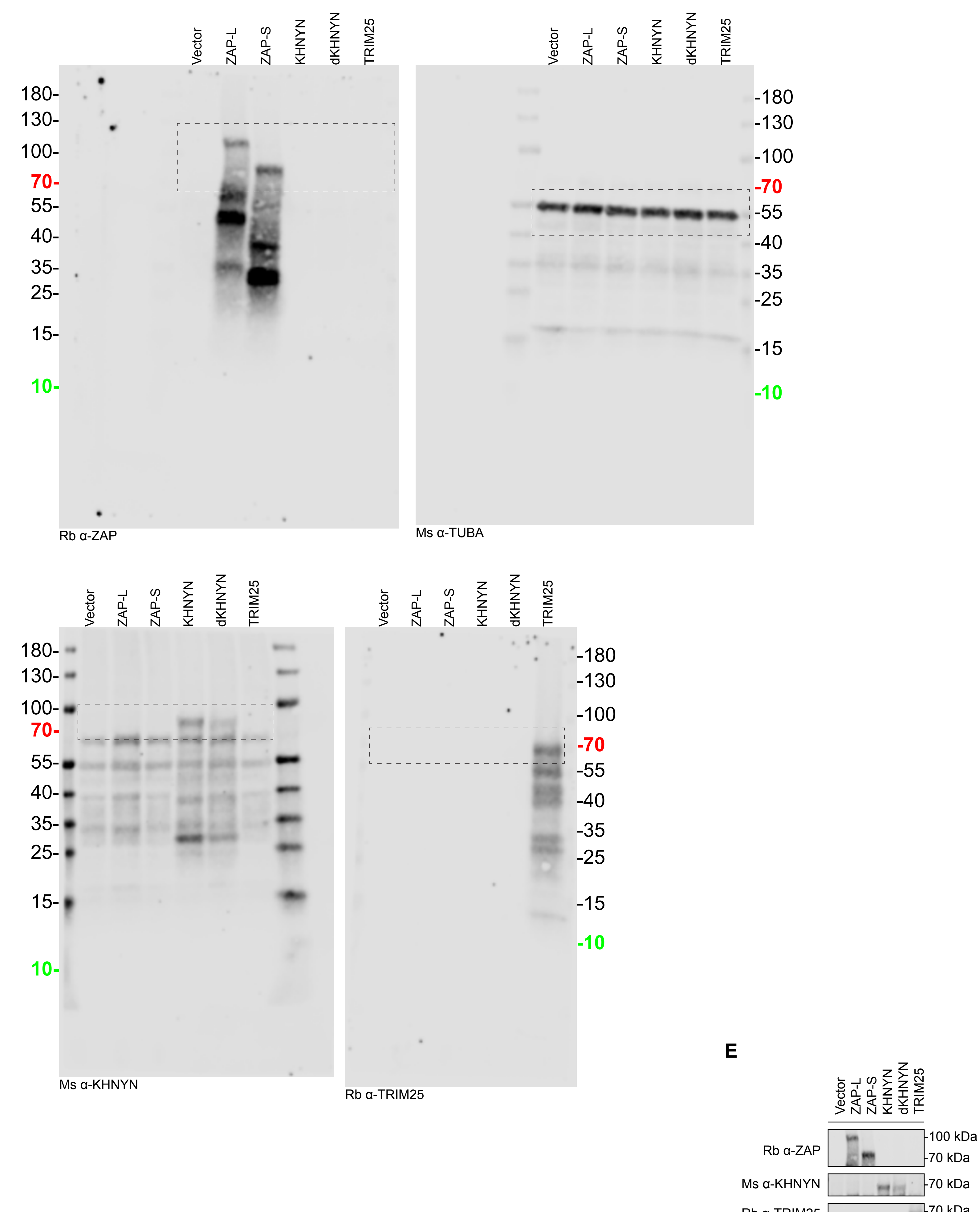


Imaged on LiCor Odyssey Fc infrared fluorescent imager
Ms primary antibodies with goat anti-mouse IgG IRDye 680LT (700nm channel: 685nm ex / 730nm em)
Rb primary antibodies with goat anti-rabbit IgG IRDye 800CW (800nm channel: 785nm ex / 830nm em)
Thermo Fisher Scientific (#26616) PageRuler Prestained Protein Ladder

Figure 2F-G

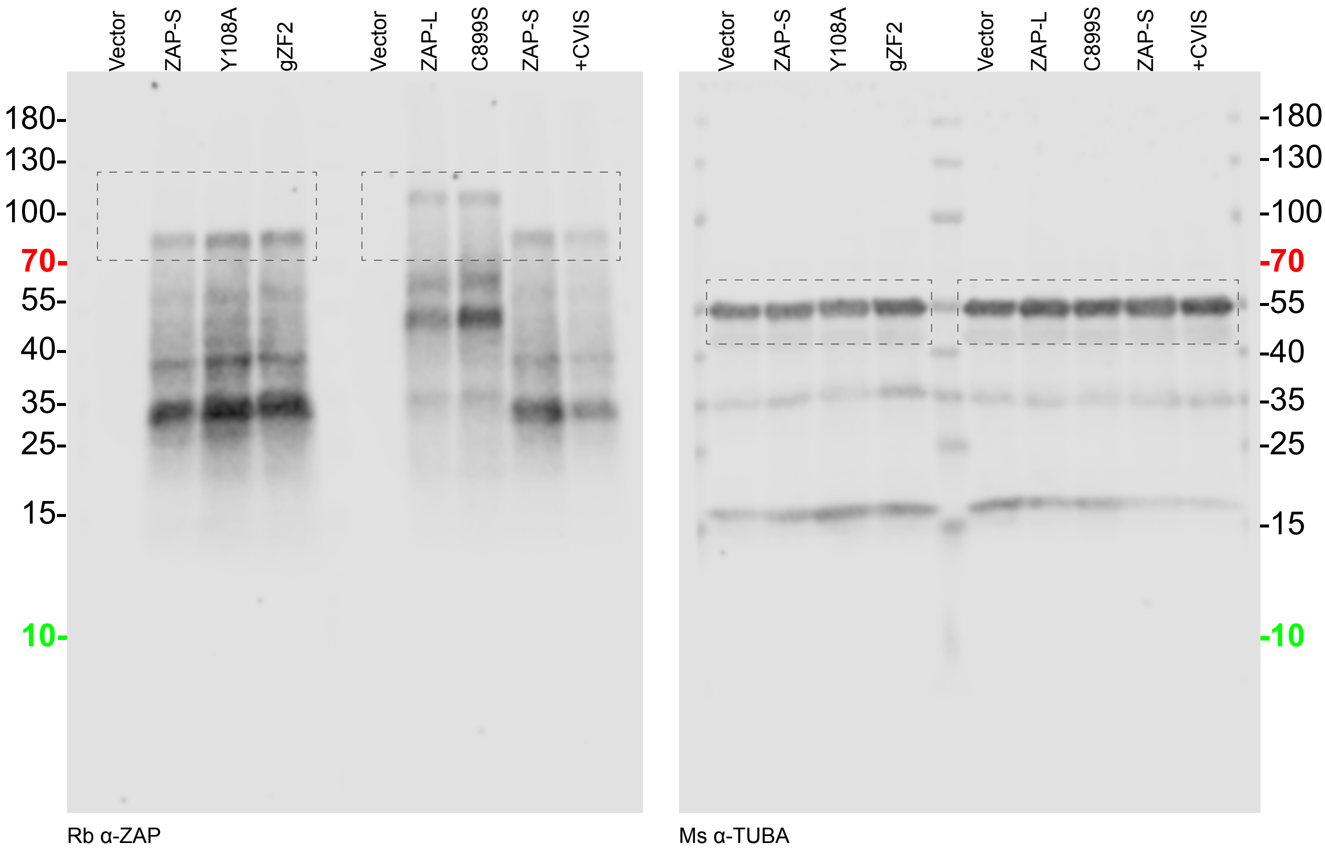


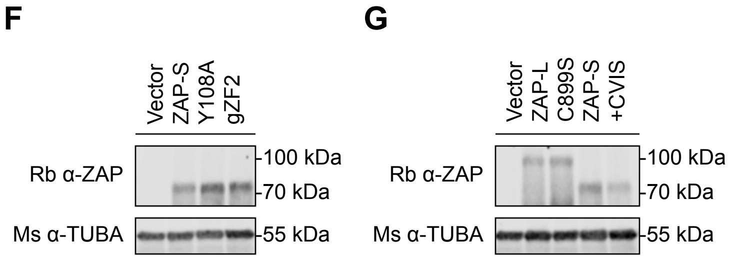


Imaged on LiCor Odyssey Fc infrared fluorescent imager
Ms primary antibodies with goat anti-mouse IgG IRDye 680LT (700nm channel: 685nm ex / 730nm em)
Rb primary antibodies with goat anti-rabbit IgG IRDye 800CW (800nm channel: 785nm ex / 830nm em)
Thermo Fisher Scientific (#26616) PageRuler Prestained Protein Ladder

Figure 2H

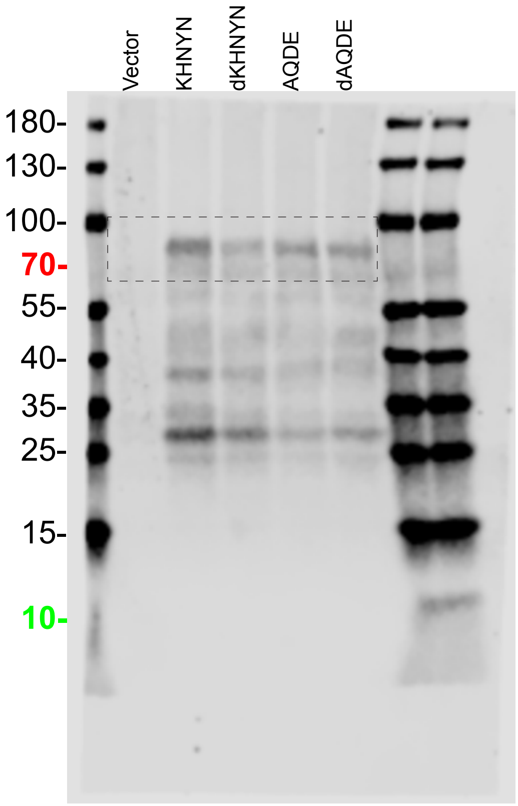


Ms α-KHNYN

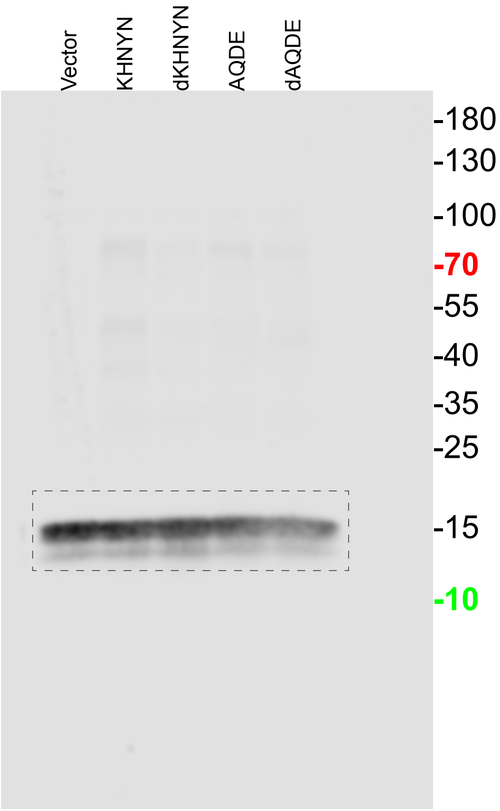


Rb α-H3

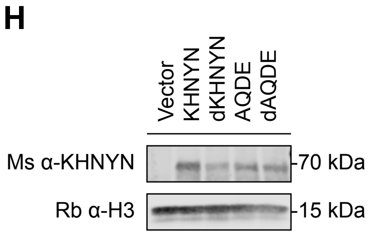


Imaged on LiCor Odyssey Fc infrared fluorescent imager
Ms primary antibodies with goat anti-mouse IgG IRDye 680LT (700nm channel: 685nm ex / 730nm em)
Rb primary antibodies with goat anti-rabbit IgG IRDye 800CW (800nm channel: 785nm ex / 830nm em)
Thermo Fisher Scientific (#26616) PageRuler Prestained Protein Ladder

Figure 3B

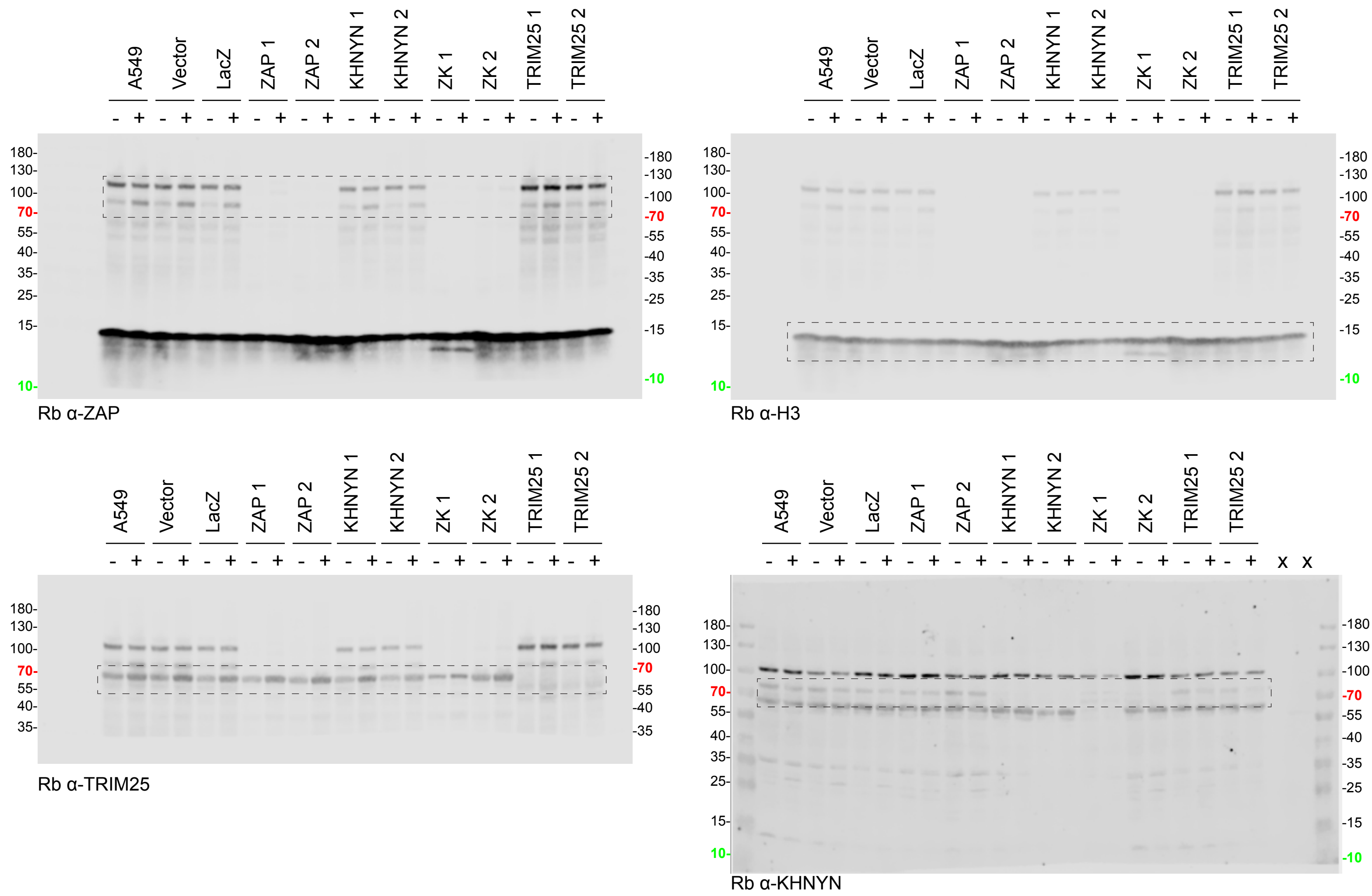


B

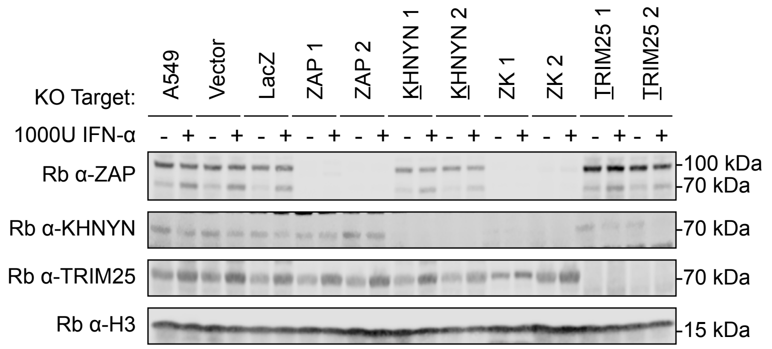


Imaged on LiCor Odyssey Fc infrared fluorescent imager
Ms primary antibodies with goat anti-mouse IgG IRDye 680LT (700nm channel: 685nm ex / 730nm em)
Rb primary antibodies with goat anti-rabbit IgG IRDye 800CW (800nm channel: 785nm ex / 830nm em)
Thermo Fisher Scientific (#26616) PageRuler Prestained Protein Ladder

Figure 4C

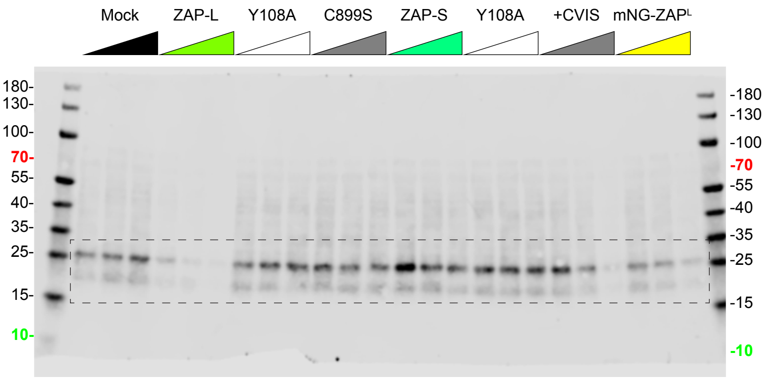


Virus Ms α-p19 (ROSV Gag p19)

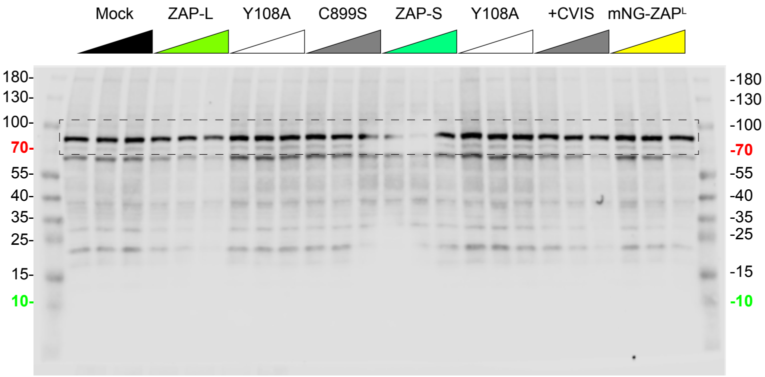


Cells Ms α-p19 (ROSV Gag Pr76)

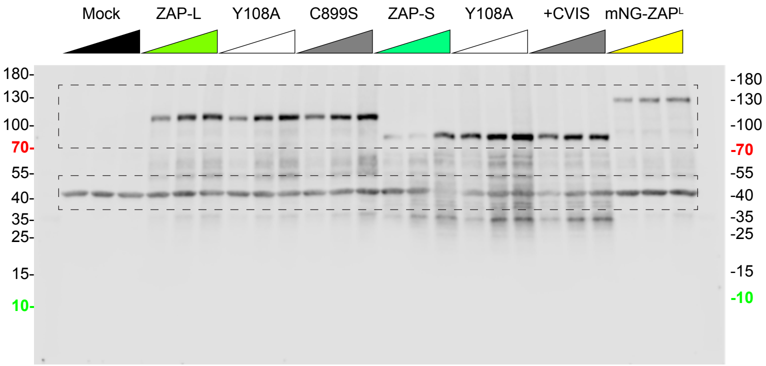


Cells Rb α-ZAP
Cells Rb α-ACTIN

Imaged on LiCor Odyssey Fc infrared fluorescent imager
Ms primary antibodies with goat anti-mouse IgG IRDye 680LT (700nm channel: 685nm ex / 730nm em)
Rb primary antibodies with goat anti-rabbit IgG IRDye 800CW (800nm channel: 785nm ex / 830nm em)
Thermo Fisher Scientific (#26616) PageRuler Prestained Protein Ladder

Figure 4D

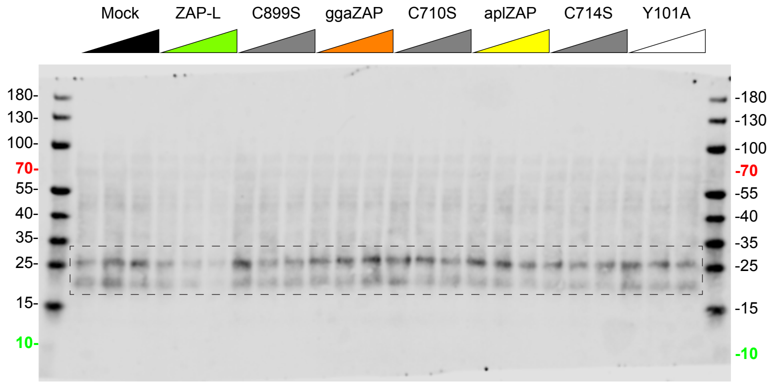


Virus Ms α-p19 (ROSV Gag p19)

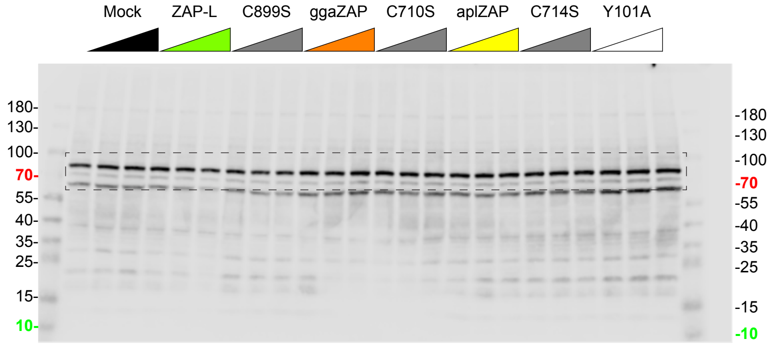


Cells Ms α-p19 (ROSV Gag Pr76)

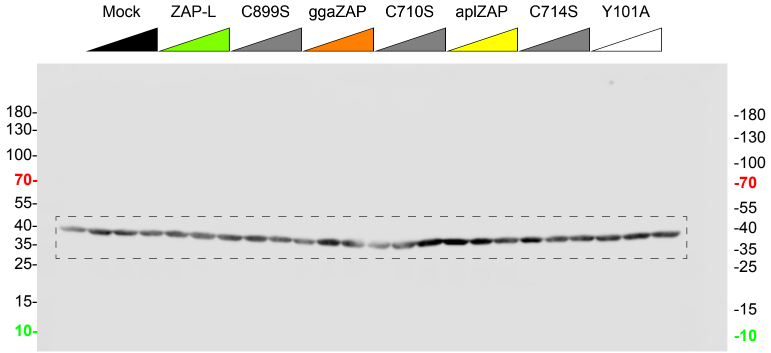


Cells Rb α-ACTIN

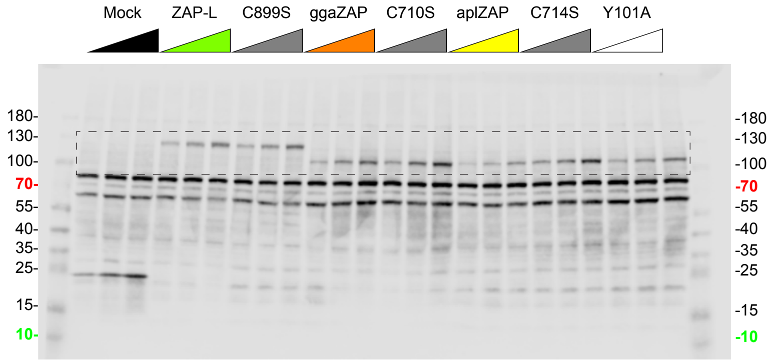


Cells Ms α-mNeonGreen

Imaged on LiCor Odyssey Fc infrared fluorescent imager
Ms primary antibodies with goat anti-mouse IgG IRDye 680LT (700nm channel: 685nm ex / 730nm em)
Rb primary antibodies with goat anti-rabbit IgG IRDye 800CW (800nm channel: 785nm ex / 830nm em)
Thermo Fisher Scientific (#26616) PageRuler Prestained Protein Ladder

Figure 4E

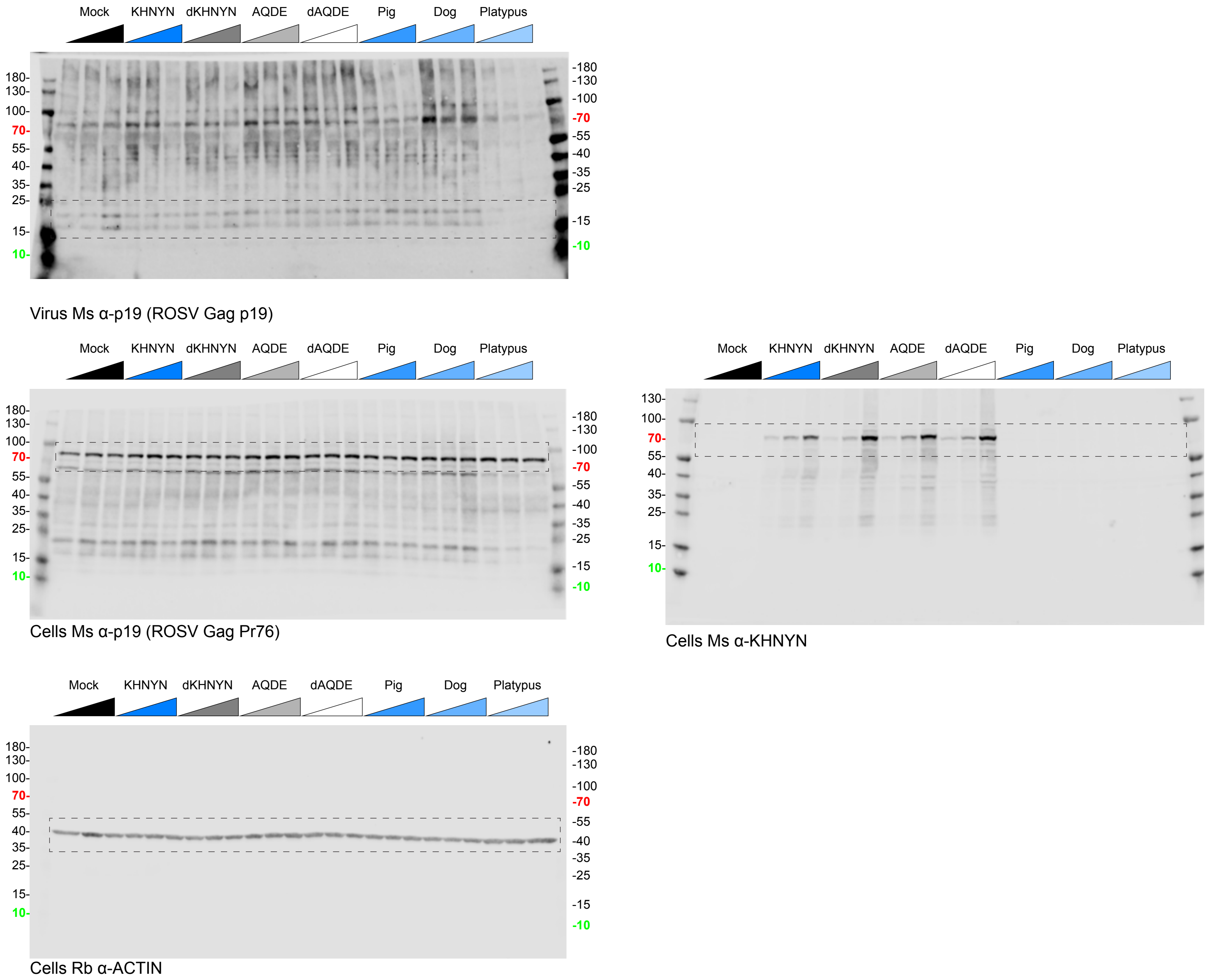


Imaged on LiCor Odyssey Fc infrared fluorescent imager
Ms primary antibodies with goat anti-mouse IgG IRDye 680LT (700nm channel: 685nm ex / 730nm em)
Rb primary antibodies with goat anti-rabbit IgG IRDye 800CW (800nm channel: 785nm ex / 830nm em)
Thermo Fisher Scientific (#26616) PageRuler Prestained Protein Ladder

S3C-D Fig

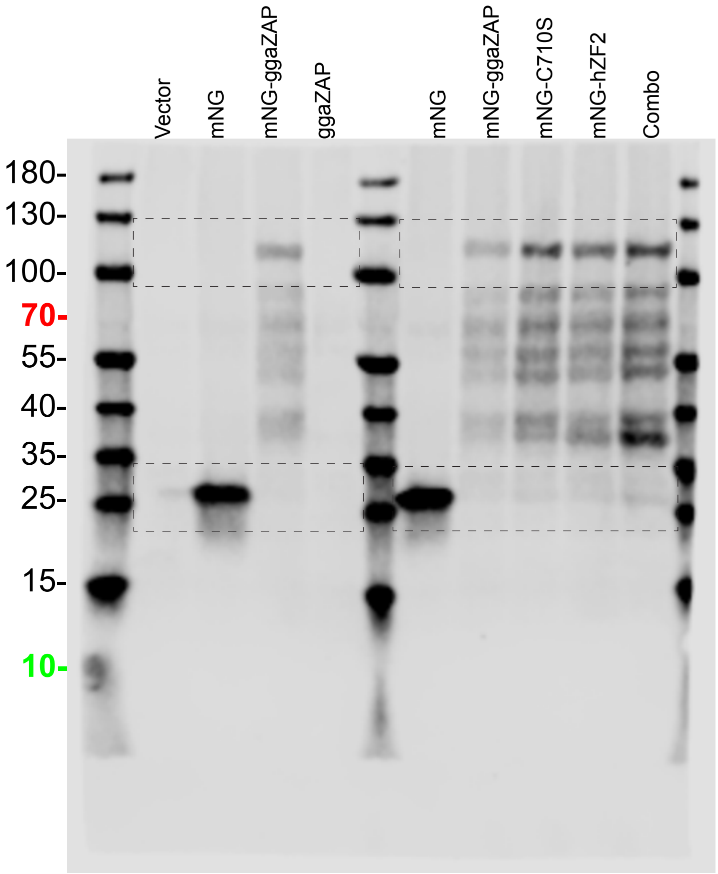


Ms α-mNeonGreen

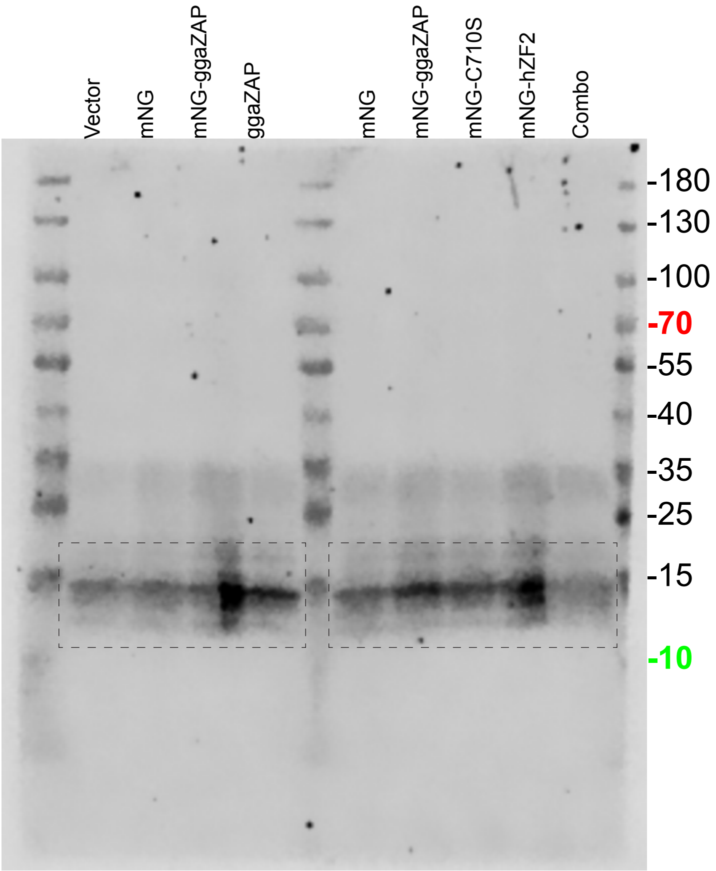


Rb α-BSD

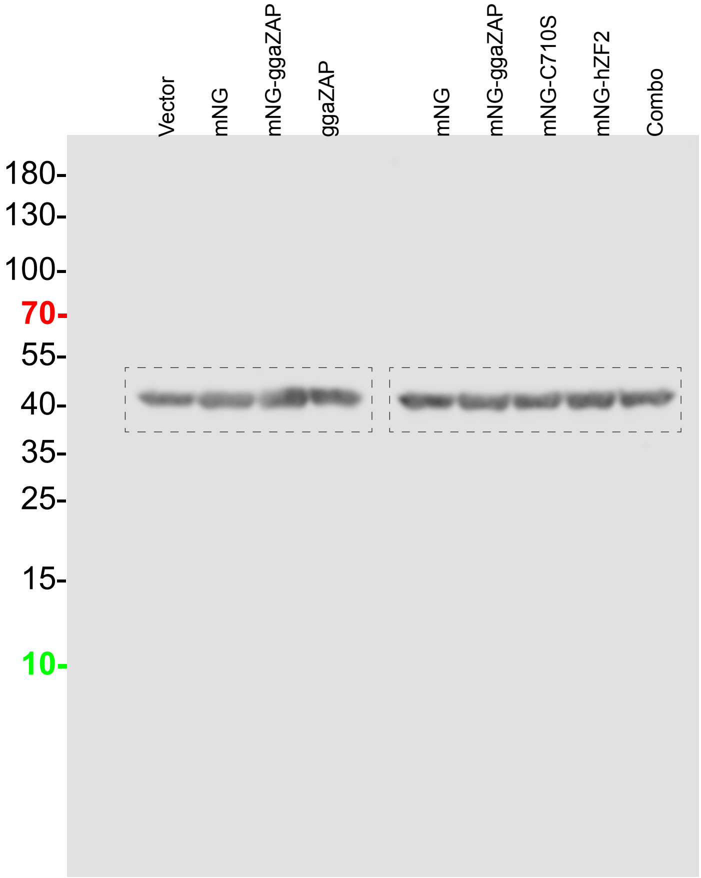


Rb α-ACTIN

**C**

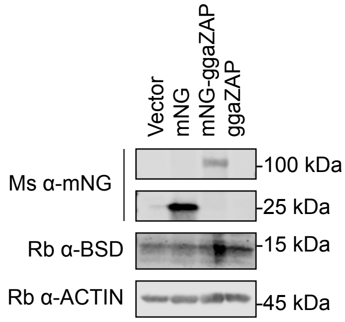


**D**

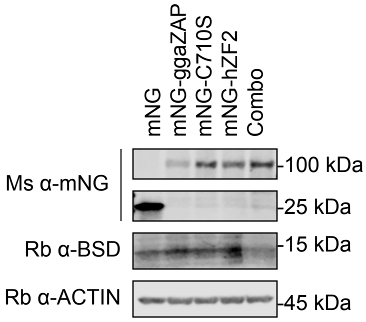


Imaged on LiCor Odyssey Fc infrared fluorescent imager
Ms primary antibodies with goat anti-mouse IgG IRDye 680LT (700nm channel: 685nm ex / 730nm em)
Rb primary antibodies with goat anti-rabbit IgG IRDye 800CW (800nm channel: 785nm ex / 830nm em)
Thermo Fisher Scientific (#26616) PageRuler Prestained Protein Ladder

S3H Fig

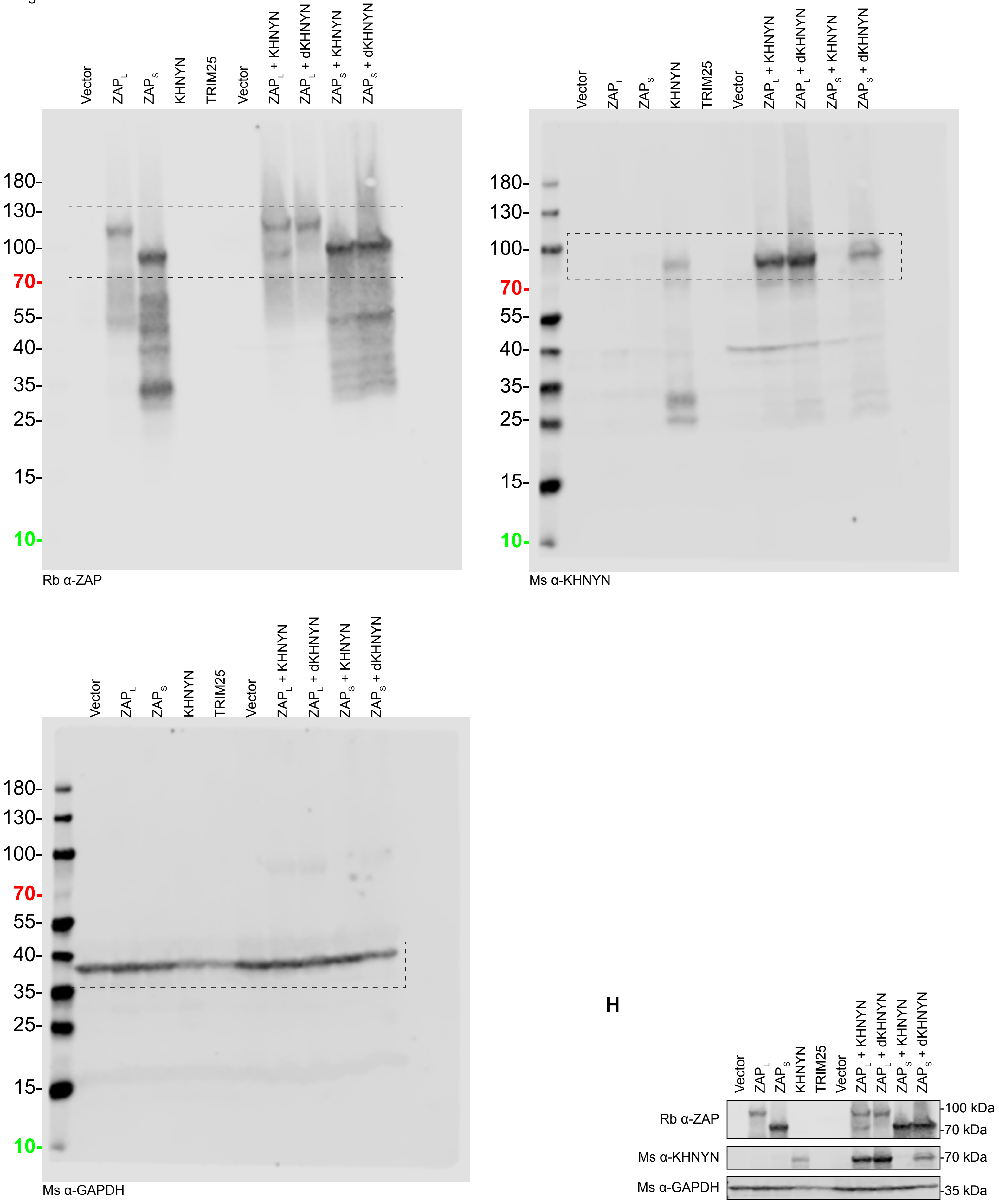


Imaged on LiCor Odyssey Fc infrared fluorescent imager
Ms primary antibodies with goat anti-mouse IgG IRDye 680LT (700nm channel: 685nm ex / 730nm em)
Rb primary antibodies with goat anti-rabbit IgG IRDye 800CW (800nm channel: 785nm ex / 830nm em)
Thermo Fisher Scientific (#26616) PageRuler Prestained Protein Ladder

S6B Fig

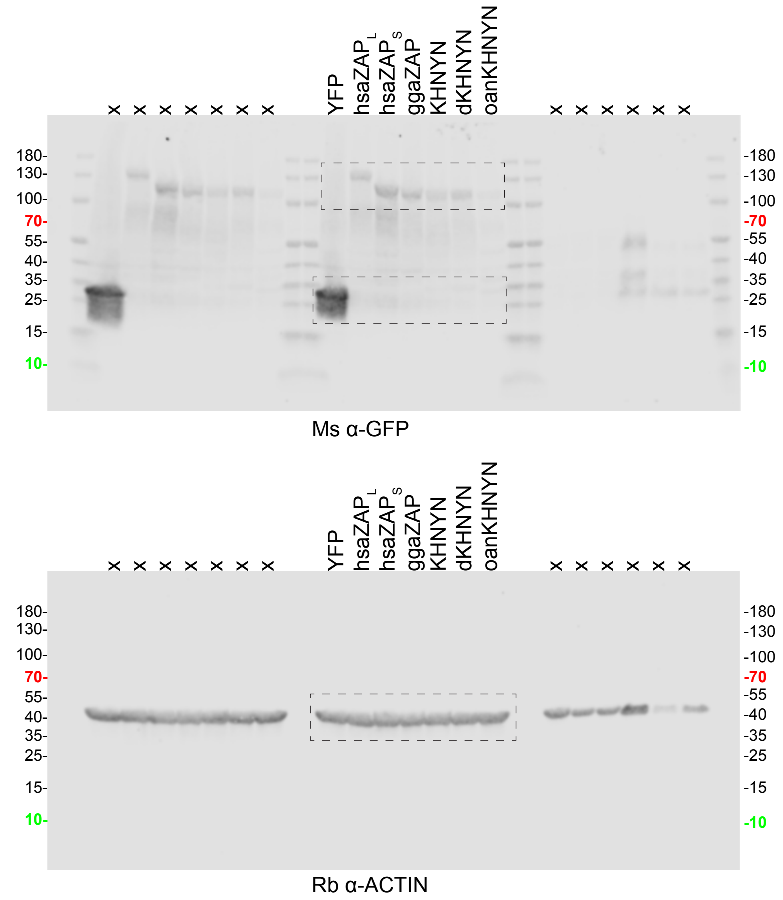


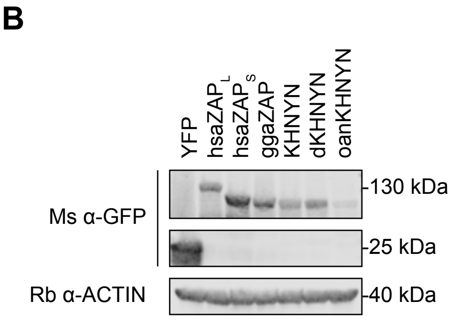


Imaged on LiCor Odyssey Fc infrared fluorescent imager
Ms primary antibodies with goat anti-mouse IgG IRDye 680LT (700nm channel: 685nm ex / 730nm em)
Rb primary antibodies with goat anti-rabbit IgG IRDye 800CW (800nm channel: 785nm ex / 830nm em)
Thermo Fisher Scientific (#26616) PageRuler Prestained Protein Ladder

S6D Fig

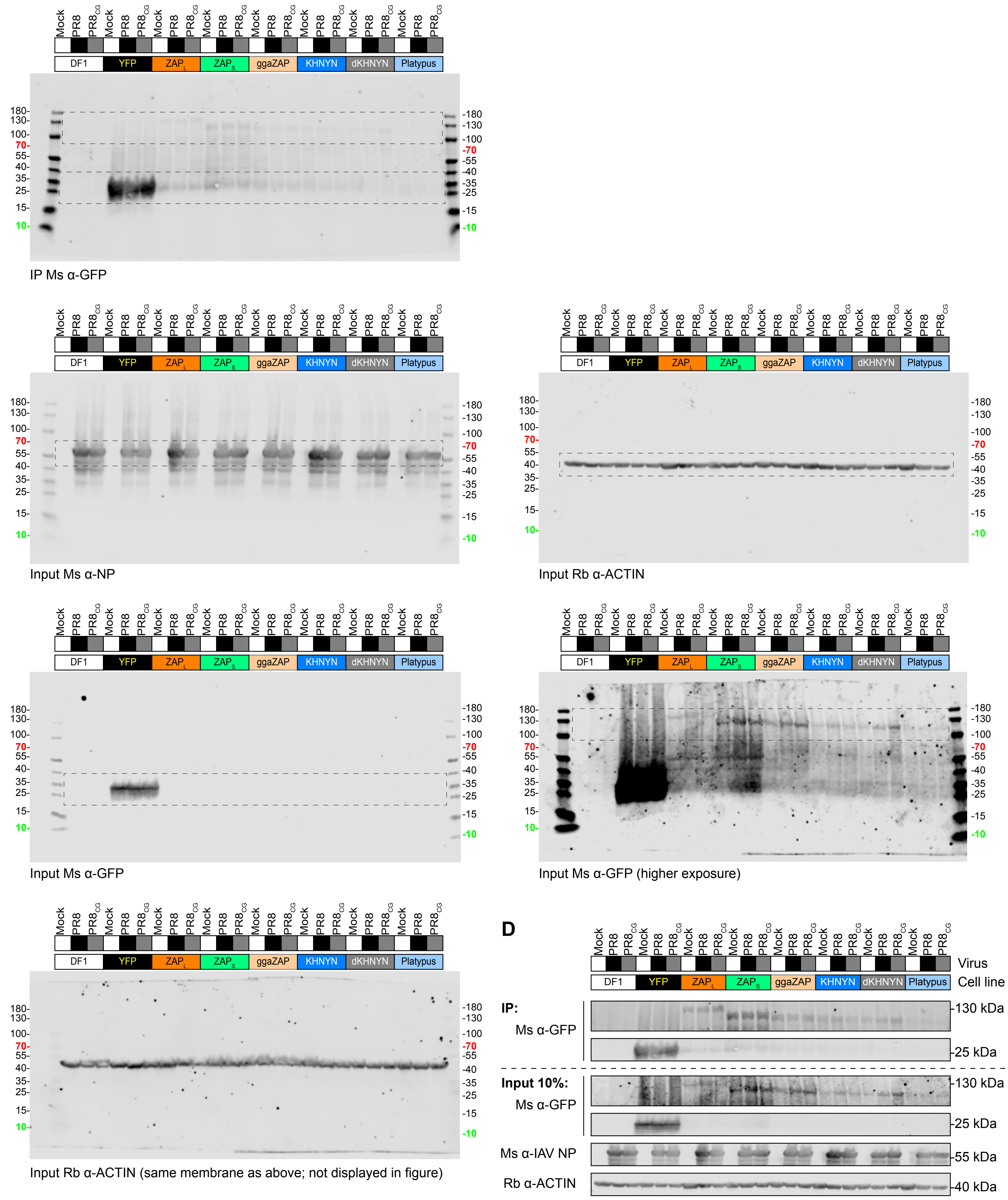


Imaged on LiCor Odyssey Fc infrared fluorescent imager

Ms primary antibodies with goat anti-mouse IgG IRDye 680LT (700nm channel: 685nm ex / 730nm em)

Rb primary antibodies with goat anti-rabbit IgG IRDye 800CW (800nm channel: 785nm ex / 830nm em)

Thermo Fisher Scientific (#26616) PageRuler Prestained Protein Ladder

S8A Fig

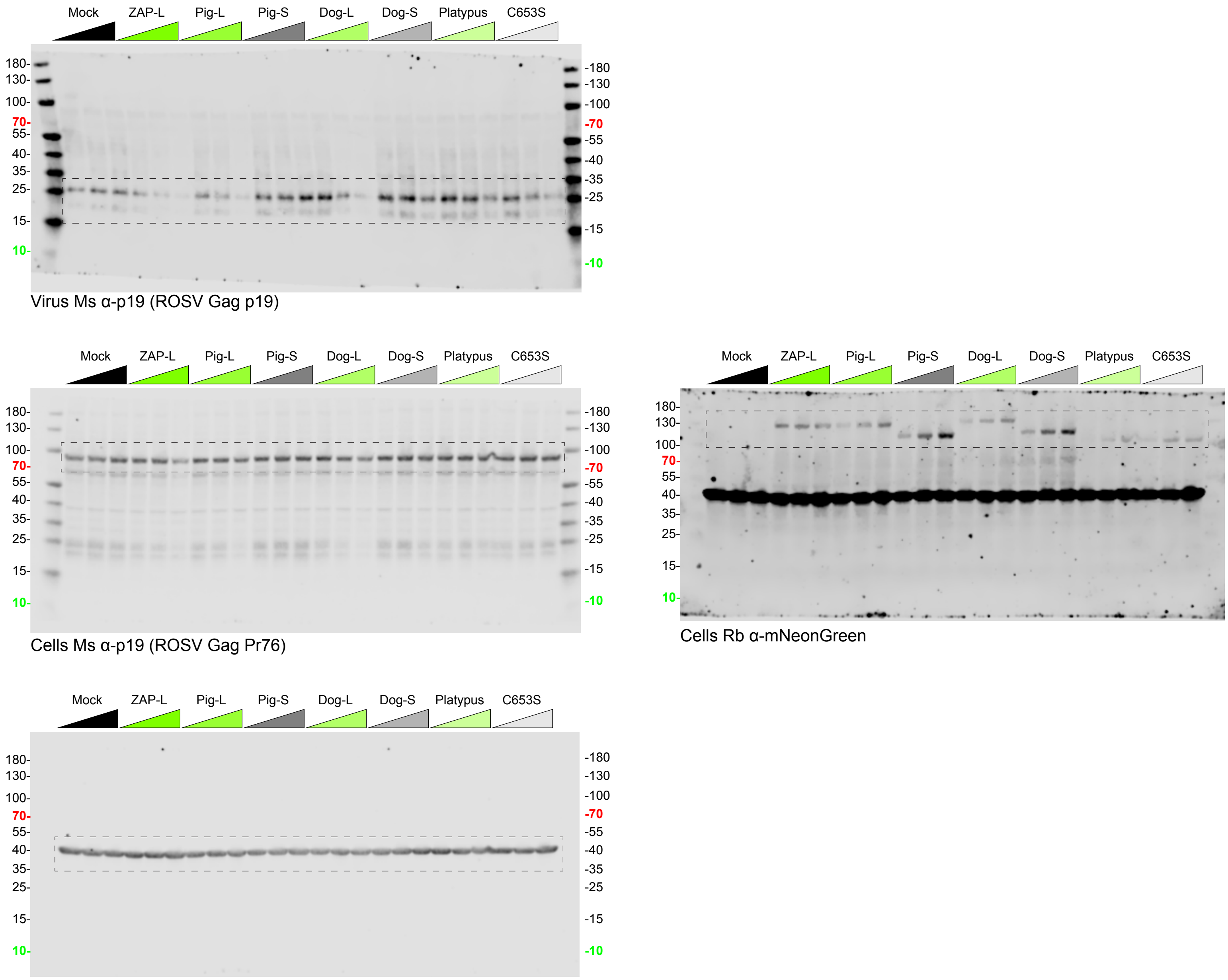


Virus Ms α-p19 (ROSV Gag p19)

Cells Ms α-p19 (ROSV Gag Pr76)

Cells Rb α-mNeonGreen

Cells Rb α-ACTIN

Imaged on LiCor Odyssey Fc infrared fluorescent imager

Ms primary antibodies with goat anti-mouse IgG IRDye 680LT (700nm channel: 685nm ex / 730nm em)

Rb primary antibodies with goat anti-rabbit IgG IRDye 800CW (800nm channel: 785nm ex / 830nm em)

Thermo Fisher Scientific (#26616) PageRuler Prestained Protein Ladder

S8B Fig

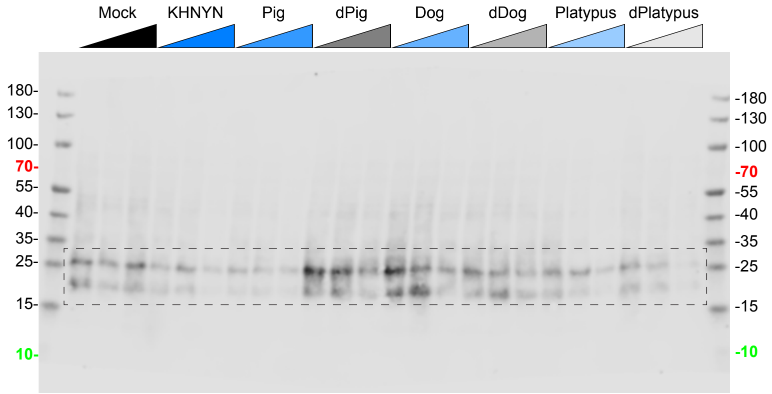

Virus Ms α-p19 (ROSV Gag p19)

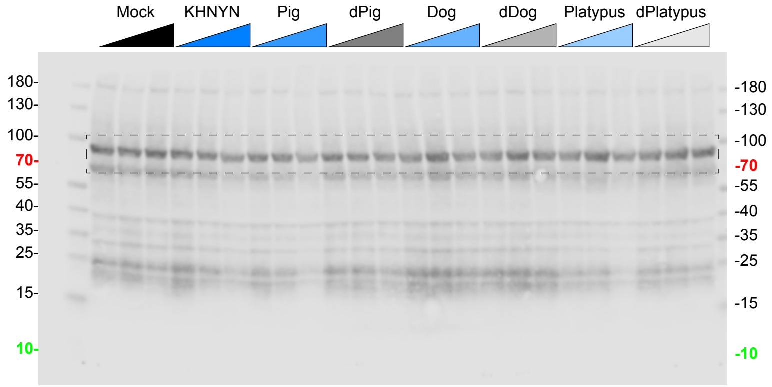

Cells Ms α-p19 (ROSV Gag Pr76)

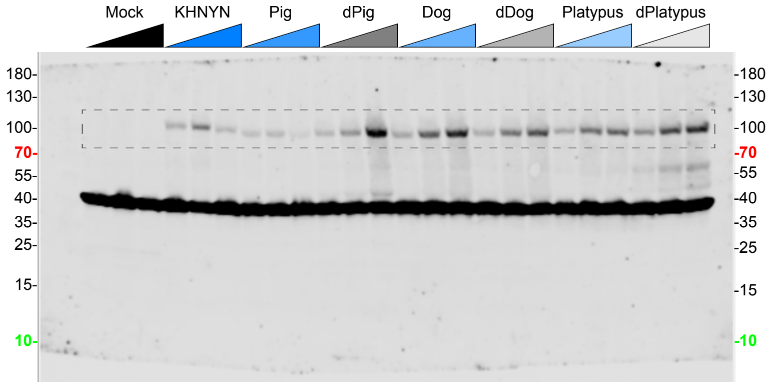

Cells Rb α-mNeonGreen

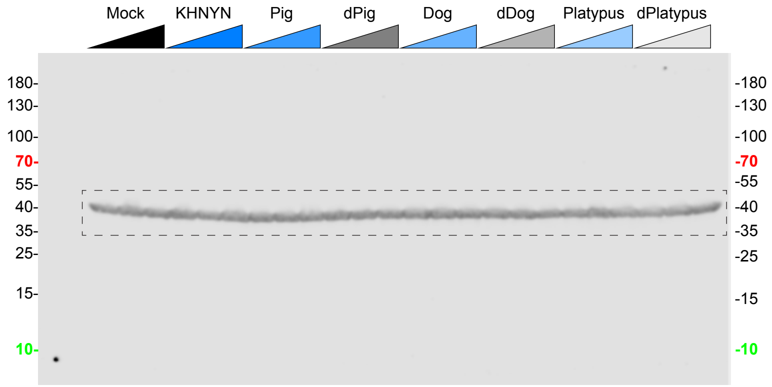

Cells Rb α-ACTIN

Imaged on LiCor Odyssey Fc infrared fluorescent imager
Ms primary antibodies with goat anti-mouse IgG IRDye 680LT (700nm channel: 685nm ex / 730nm em)
Rb primary antibodies with goat anti-rabbit IgG IRDye 800CW (800nm channel: 785nm ex / 830nm em)
Thermo Fisher Scientific (#26616) PageRuler Prestained Protein Ladder

S8C Fig

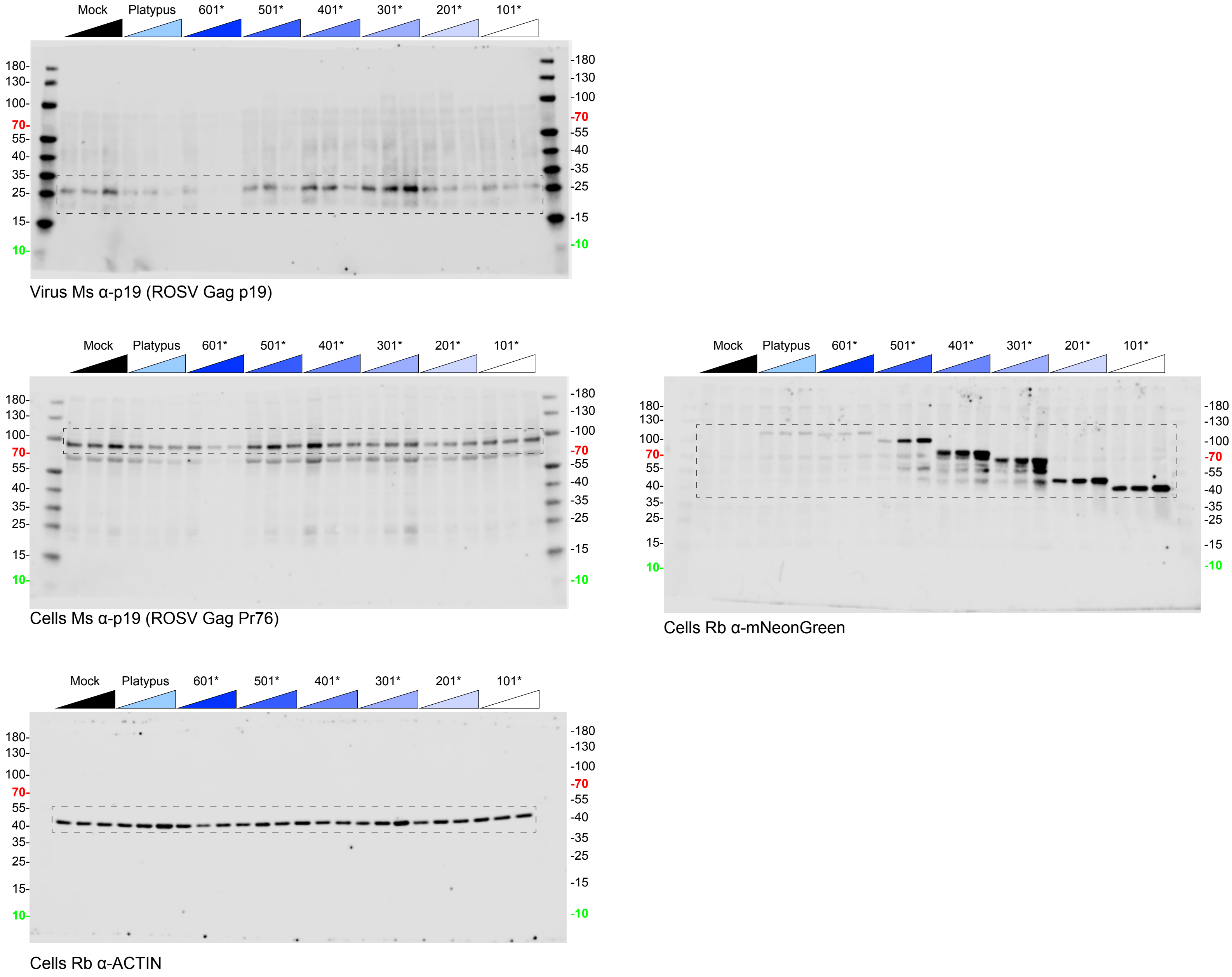


Imaged on LiCor Odyssey Fc infrared fluorescent imager
Ms primary antibodies with goat anti-mouse IgG IRDye 680LT (700nm channel: 685nm ex / 730nm em)
Rb primary antibodies with goat anti-rabbit IgG IRDye 800CW (800nm channel: 785nm ex / 830nm em)
Thermo Fisher Scientific (#26616) PageRuler Prestained Protein Ladder

S8D Fig

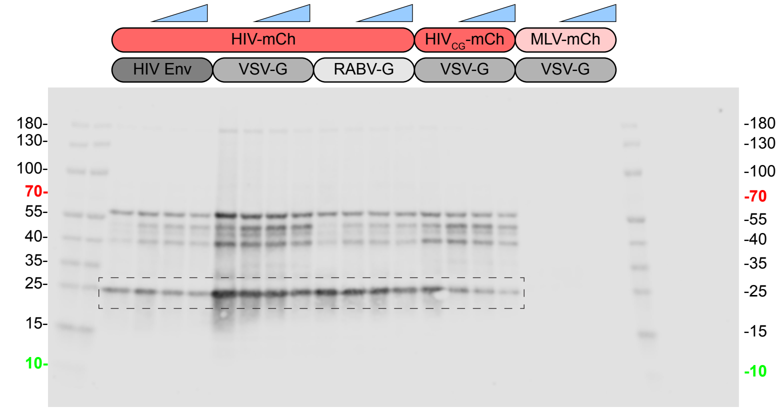


Virus Ms α-p24 (HIV-1 Gag p24)

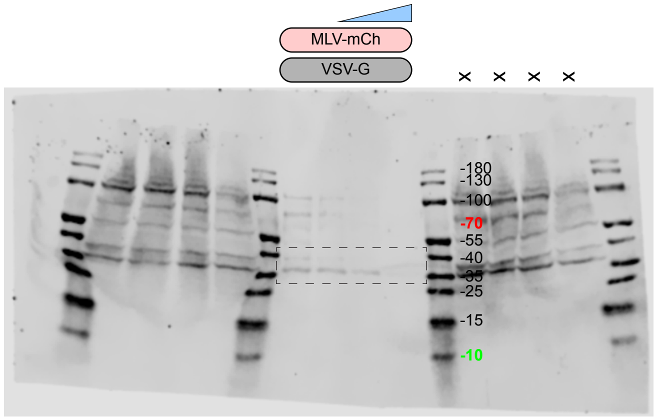


Virus Ms α-HA epitope (MLV Gag-HA-mCherry)

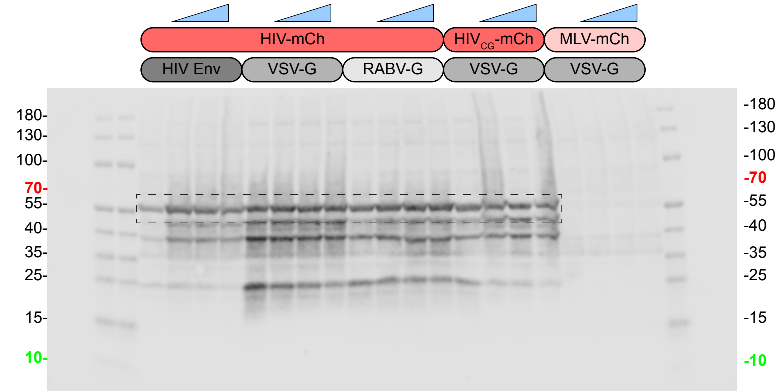


Cells Ms α-p19 (ROSV Gag Pr76)

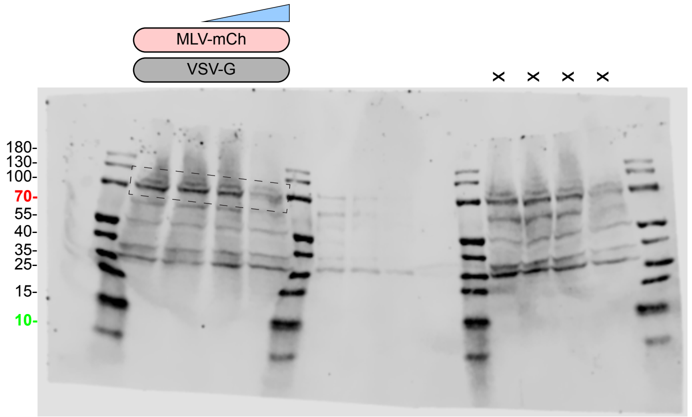


Cells Rb α-HA epitope (MLV Gag-HA-mCherry)

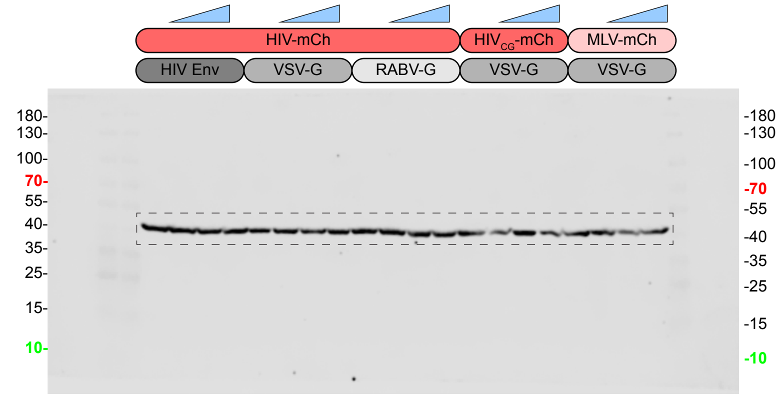


Cells Rb α-ACTIN

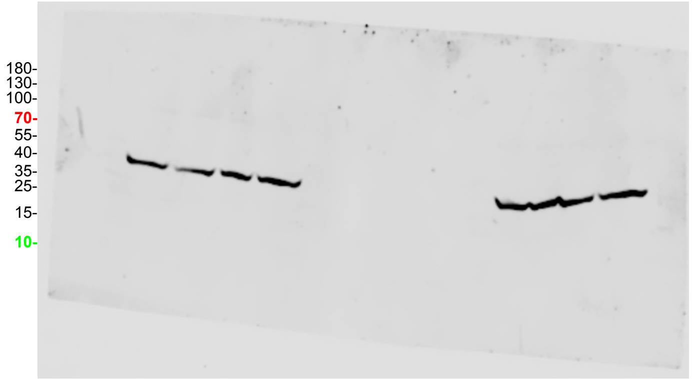


Cells Rb α-ACTIN (same membrane as above, not displayed in figure)

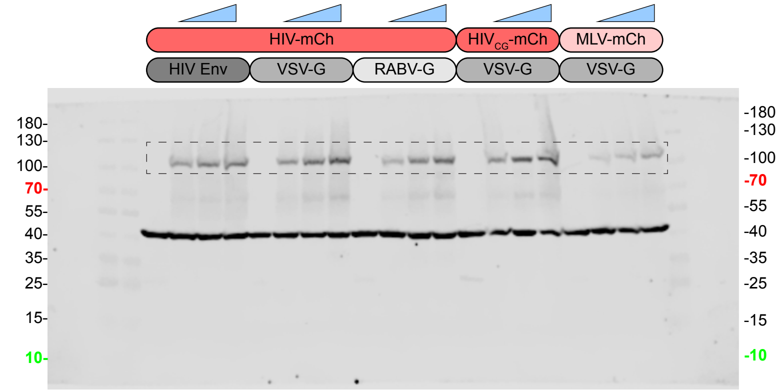


Cells Rb α-mNeonGreen

Imaged on LiCor Odyssey Fc infrared fluorescent imager
Ms primary antibodies with goat anti-mouse IgG IRDye 680LT (700nm channel: 685nm ex / 730nm em)
Rb primary antibodies with goat anti-rabbit IgG IRDye 800CW (800nm channel: 785nm ex / 830nm em)
Thermo Fisher Scientific (#26616) PageRuler Prestained Protein Ladder

S8E Fig

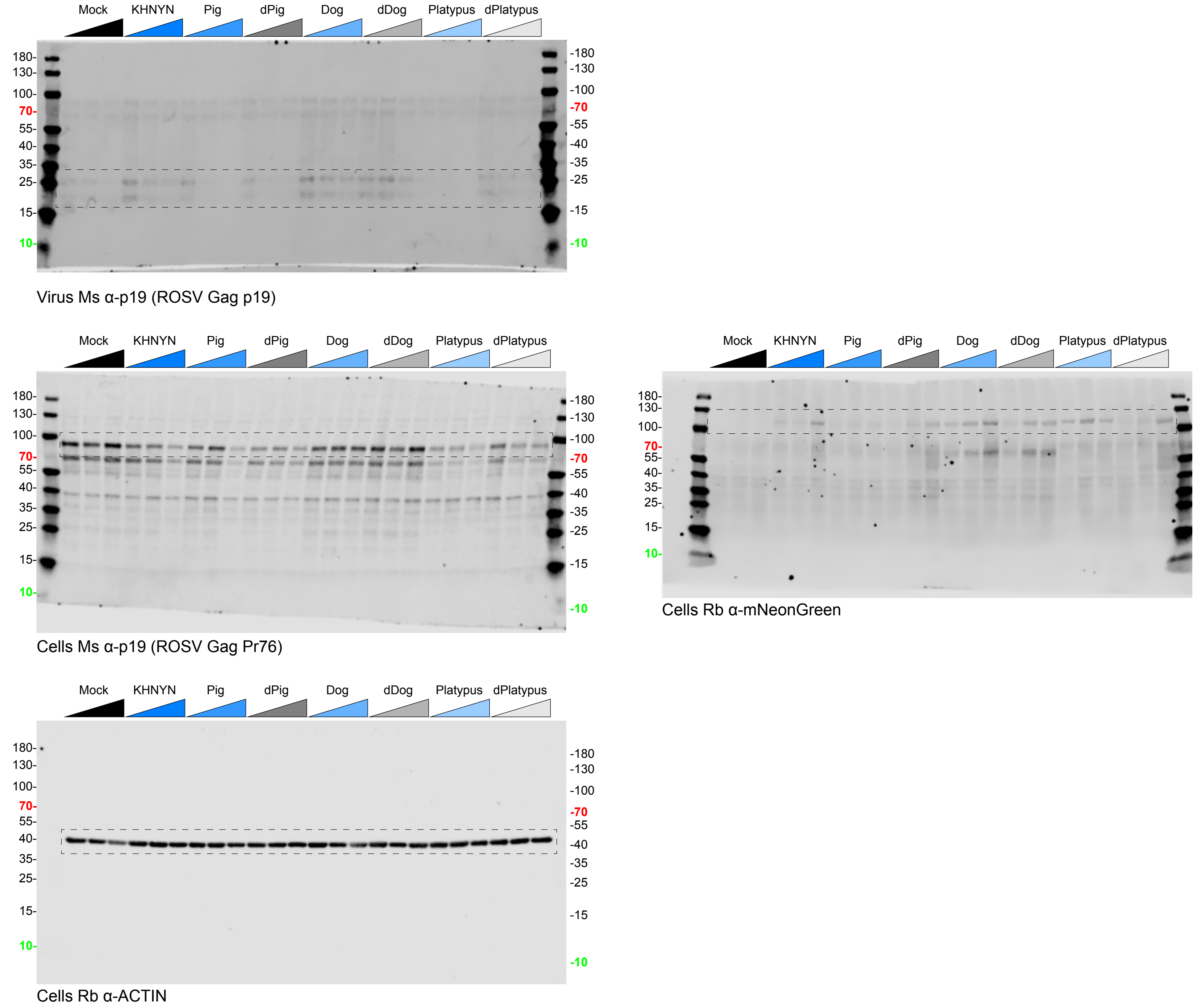


Imaged on LiCor Odyssey Fc infrared fluorescent imager
Ms primary antibodies with goat anti-mouse IgG IRDye 680LT (700nm channel: 685nm ex / 730nm em)
Rb primary antibodies with goat anti-rabbit IgG IRDye 800CW (800nm channel: 785nm ex / 830nm em)
Thermo Fisher Scientific (#26616) PageRuler Prestained Protein Ladder
