# Supplementary material for: Mammalian antiviral proteins ZAP and KHNYN can independently restrict CpG-enriched avian viruses
Source: PLoS Biol. 2025 Oct 28;23(10):e3003471. doi: 10.1371/journal.pbio.3003471 (PMC12582503; doi:10.1371/journal.pbio.3003471)
Supplement: Attachment — Submitted filename: Becker ZKT Response PBIO V4.docx [file pbio.3003471.s021.docx]

------------------------------------
EDITOR CORRESPONDENCE

------------------------------------

Dear Dr Becker,

Thank you for your patience while your manuscript "Mammalian ZAP and KHNYN independently restrict CpG-enriched avian viruses" was peer-reviewed at PLOS Biology. It has now been evaluated by the PLOS Biology editors, an Academic Editor with relevant expertise, and by several independent reviewers.

In light of the reviews, which you will find at the end of this email, we would like to invite you to revise the work to thoroughly address the reviewers' reports. As you will see below, majority of reviewers are positive about the relevance and novelty of the study, yet some concerns have raised during revision. Reviewer 1 acknowledges the potential importance of the study but suggests that several experiments are lacking necessary controls. Reviewer 2 agrees that the study tackles an important question, but points out the absence of functional validation, specifically the lack of direct evidence for ZAP and KHNYN RNA binding/degradation, as well as a mechanistic explanation for how platypus KHNYN inhibits avian viruses. Reviewer 3 highlights that some statements in the manuscript should be toned down, particularly those based on results from lab-adapted PR8 and PR8cg strains.

Important: After discussions with the Academic Editor and the reviewers, we do not expect you to provide mechanistic insights into how platypus KHNYN functions. However, we do require you to provide biochemical or functional validation demonstrating that KHNYN can inhibit IAV independently of ZAP. Specifically, this should involve testing whether KHNYN can directly bind to or degrade RNA in the absence of ZAP expression. Additionally, we ask that you include the controls suggested by Reviewer 1 and either modify or tone down the general statements highlighted by Reviewer 3. Alternatively, testing additional IAV strains may help support such claims. Other concerns raised can be addressed through further clarification or modifications in the text.

We appreciate the collaborative discussions among editors and reviewers. We detail our responses to those suggestions as well as specific reviewer comments. We provide evidence for an interaction between human KHNYN and IAV RNA (independent of human ZAP) by immunoprecipitation-qPCR in **S6D Fig**. We also provide evidence for an interaction between ZAP-S and IAV RNA. We also validate independent KHNYN function in the absence of chicken ZAP by restricting ROSV in **S8E Fig** (in chicken ZAP knockout DF1 cells).

In addition, by providing western blots for **Fig 4D** and **S8B Fig** we observed a dose-dependent inhibition of retroviral virion assembly and release by platypus KHNYN without drastically affecting intracellular expression of Gag. We also performed IAV minigenome assays that suggest mammalian KHNYN may negatively impact viral polymerase activity in **Fig 4G**. These provide initial insights into antiviral mechanisms of platypus KHNYN. We also highlight future directions to investigate platypus KHNYN antiviral mechanisms.

In addition to mollifying our language as suggested by Reviewer 3, we tested an avian IAV strain (OH175) using chicken DF1 cells expressing YFP-tagged human ZAP-S and KHNYN in **S6A-B Figs** showing modest restriction – perhaps related to the modest (but natural) CpG enrichment of this strain (shown in **S6C Fig**). We also highlight that we initially tested human pandemic H1N1 Cal04 and OH175 in single cycle infections for restriction by platypus KHNYN in **Fig 4F**. Finally, we added language to our discussion/limitations that highlights the need for future testing of multiple influenza strains derived from multiple host species for restriction by ZAP and KHNYN orthologues. We believe this strikes an appropriate balance in highlighting our novel findings using PR8 and PR8_CG_ while providing more data with an avian virus strain.

In summary, we addressed the consensus suggestions from the collaborative review team as well as specific comments from Reviewer 1 and Reviewer 3. In so doing, we also provided a balanced attempt at addressing comments from Reviewer 2 within the scope of this current study. Together, we believe this improves our manuscript greatly and provides more mechanistic data regarding the ZAP-independent mechanisms of human and platypus KHNYN antiviral activity. Please find more detailed responses and quoted lines below.

------------------------------------
REVIEWERS' COMMENTS:
------------------------------------

Reviewer #1:

In this manuscript the authors demonstrate that avian strains of IAV contain significantly more CpG content and demonstrate avian ZAP and all iso-forms are incapable of reducing viral replication by observing this phenomenon for chicken. The authors then went on to establish several mammalian ZAP and KHNYN can independently reduce viral replication of CpG enriched IAV and ROSV by evaluating viral titers in chicken cells via multicycle replication assays. Specifically, they demonstrated that human ZAP-S and KHNYN reduce viral replication for CpG rich IAV, while ZAP-L reduced ROSV (avian virus) replication. They demonstrated that other mammalian CpG dependent restriction mechanisms (i.e platypus) can reduce virus replication and that ZAP and KHNYN can function independently. Though a mechanism was not thoroughly established, this work provides the foundation research on the independent antiviral capabilities of KHNYN and characterization of the mechanism. These studies could have important implications and impact in the field by advance our understanding on mechanisms of viral evolution, including the role of KHNYN in reducing CpG rich IAV replication, new anti-viral mechanism that is specific for CpG enriched virions in mammals, and species-specific roles of ZAP. There some minor issues with controls lacking, as described below

We appreciate the kind summary and acknowledgement that our work represents an exciting potential new direction in ZAP and KHNYN antiviral restriction mechanisms.

Major comments:
1. Figure 1D 1E and 1F show overexpression of ggaZAP with PR8 or PR8CG, and no differences are observed in titers as compared to vector or mNeogreen. The authors need to show that there is equal expression of proteins. Otherwise the comparison can not be made. In addition, a positive control needs to be included as comparison. For example human ZAP should inhibit PR8CG ? or another antiviral factor to show inhibition as control.

We appreciate the comment and have now included western blots detecting mNG as well as blasticidin S deaminase (BSD) to show relatively similar expression of transduced genes for **Figs 1D-F** in new **S3C-D Figs**. There is no commercially available antibody for untagged chicken ZAP. However, the retroviral transduction vector used for expressing proteins of interest (e.g. empty vector, mNG, mNG-ggaZAP, or untagged ggaZAP) also encodes BSD downstream of an IRES on a bicistronic mRNA. We now also include western blots for mNG, mNG-tagged chicken ZAP, or tagged mutants that further confirms expression across conditions used in **Fig 1F** (see **S3D Fig**) of BSD as well as mNG-tagged chicken ZAP).

Line 168: “We confirmed expression of transduced proteins by western blot for mNG as well as blasticidin S deaminase (BSD)[48, 49] expressed on the bicistronic mRNA and used for drug selection (**S3C Fig**).”

Line 180: “We confirmed expression of transduced stably expressed proteins by western blot for mNG as well as BSD (**S3D Fig**).”

We understand the reviewer’s comment regarding a positive control. When initially performing the experiments, we did not necessarily know that human ZAP would restrict PR8 or PR8_CG_ in this context or if it would be an appropriate positive control for viral restriction. Yet, in **Fig 2**, we show that human ZAP-S restricts PR8_CG_ relative to Vector transduced cells – the same Vector transduced stable cells used in **Fig 1D**. We have added language to clarify that these are the same cells to allow for relative comparisons across those figures. We also provide western blot data showing expression of these chicken ZAP proteins and controls used in **Fig 1** (see **S3C-D Figs**) as well as human proteins expressed in **Fig 2** (see **Fig 2E-H**). In addition, we included new data that compares PR8, PR8_CG_, and avian OH175 replication in cells stably expressing YFP, YFP-tagged human ZAP-L, ZAP-S, KHNYN, and dKHNYN to YFP-tagged chicken ZAP in **S6A-B Figs**. Those experiments synthesize results across **Figs 1 and 2** including chicken and human ZAP proteins directly compared.

Line 245: “The Vector transduced cells used in **Fig 1D** showing chicken ZAP had minimal, if any, effects on PR8 or PR8_CG_ are the same Vector transduced cells used throughout **Fig 2A-D** showing human ZAP-S and KHNYN restricted PR8_CG_ allowing for relative comparisons in ability for these proteins to restrict these IAV. Nonetheless, we wanted to compare human ZAP-S restriction of PR8 or PR8_CG_ to chicken ZAP directly in multicycle IAV replication assays. Using chicken DF-1 cells stably expressing YFP-tagged proteins, we found that human ZAP-S and KHNYN restricted PR8_CG_ while human ZAP-L, chicken ZAP, and dKHNYN failed to negatively affect PR8 or PR8_CG_ (**S6A-B Fig**). Human ZAP-S and KHNYN also modestly restricted an avian IAV strain (OH175) with a modest but natural CpG-enriched PA segment (**S6A-C Fig**).”

2. The same comment about control for Figures 4C,D,E. expression of proteins transfected need to be shown. If good antibodies are not available, at least other ways to show expression should be attempted (RNA expression, tagged proteins, etc).

We appreciate the comment and have included western blots of proteins from transfected plasmids detecting cellular expression as well as purified virions in **Fig 4**. We also included western blots for **S8 Fig**. We note the cellular expression of retroviral Gag proteins and loading controls were largely consistent across conditions and proteins of interest (e.g. ZAP or KHNYN) exhibited dose-dependent expression as expected.

Line 346: “We detected cellular expression of retroviral (ROSV Gag^Pr76^ and Gag^p19^; HIV-1 Gag^Pr55^ and Gag^p24^; MLV Gag^mCh^) and antiviral proteins for retroviral assembly and single-cycle infectivity assays as well as loading controls and purified virions from culture supernatants.”

3. Though they do present the double knock out, ZK1 and ZK2, they should concurrently express ZAP-S and KHNYN to confirm a decrease in viral replication that is more significant than the independent phenomenon as KHNYN is a co-factor of ZAP.

We appreciate the comment and note that in these contexts, we show that ZAP and KHNYN are not additive or necessarily synergistic supported by the independent activity shown in **Fig 2** when individually overexpressed as well as in failure of single knockouts in human A549 cells to increase replication in **Fig 3**. If these were synergistic, we would have expected a single knockout to yield some increase to replication and double knockout to further increase that effect. We did not observe this and highlight these multiple restriction possibilities in the discussion.

Line 492: “In addition, ZAP might restrict some sequences, KHNYN might restrict other sequences, and they may act together against yet a third group of sequences. ZAP and KHNYN may also exhibit overlapping and independent restriction preferences as shown in (**Fig 2-3**).”

Constitutive overexpression of both human proteins in chicken DF1 cells proved to be technically challenging. Specifically, following sequential or simultaneous transduction of DF1 cells with ZAP-S and either KHNYN or dKHNYN – we found that KHNYN was not readily detectable by western blot while dKHNYN was detectable but lower than in combination with ZAP-L (**S3H Fig**). Those attempts used the natural human coding sequences for ZAP and KHNYN. Furthermore, we now highlight future directions that might use inducible systems and/or codon-optimized ZAP or KHNYN constructs in our discussion/limitations.

Line 187: “Attempts at co-expression of human ZAP-S and KHNYN as exogenous transgenes in chicken DF-1 cells were complicated by apparent ZAP-S related inhibition of KHNYN protein expression or stability (**S3H Fig**).”

Line 532: “We attempted to build chicken DF-1 cells constitutively expressing both ZAP and KHNYN using native human CDS. Future work may benefit from codon-optimized and inducible expression systems compared across different host species.”

Minor Comments:
1. The schematic (Figure 2A), while visually appealing, does not effectively convey a clear message.

We appreciate the aesthetic compliment and removed the schematic.

2. Define ZK1 and ZK2 in the body of the text and/or figure legend

We apologize for this oversight, appreciate the comment, and added definitions for ZK1-2 as needed.

Line 289: “Therefore, we tested multicycle replication of PR8 and PR8_CG_ in double knockout (ZAP and KHNYN depleted, ZK1-2) cells revealing that genetic loss of both ZAP and KHNYN increased replication of PR8_CG_ relative to parental A549 or Vector cell lines (**Fig 3D**) supporting the hypothesis that ZAP and KHNYN can independently restrict of CpG enriched IAV.”

3. Make a note that CpG rich IAV replicates less or is somewhat attenuated compared to the original IAV strain.

We appreciate this comment and now note that PR8_CG_ is attenuated relative to PR8 as observed in previous publications (Gaunt, et al., 2016, PMID: 26878752).

Line 167: “PR8_CG_ exhibits consistently lower replication relative to wild-type PR8 as previously shown[47] but allows comparisons for viral replication across different cellular contexts.”

------------------------------------

Reviewer #2:

Influenza A virus (IAV) represents a significant threat to global health, given its cross-species transmission and the antiviral mechanisms that suppress IAV zoonoses are poorly understood. Previous studies have interrogated the host proviral factors involved in the cross-species interface at the amino acid and protein structure level but have not elucidated the network of restriction factors that target zoonotic IAV infections nor any viral nucleic acid adaptations driven by host-pathogen interactions during infections. Mammalian zinc finger antiviral protein (ZAP) has been shown by other groups to restrict zoonotic IAV due to ZAP's CpG-targeting activity. This study demonstrates that CpG dinucleotide suppression is a defining feature of mammalian-adapted IAV and may be driven by host restriction factors such as ZAP and KHNYN. Using comparative genomics and structural modeling, the authors show that avian IAV maintains higher CpG content, potentially due to the absence of a KHNYN orthologue and structural divergence in avian ZAP that may impair CpG recognition. Functional assays in avian cells reveal that human ZAP-S and KHNYN can independently restrict CpG-enriched IAV, while chicken ZAP fails to restrict IAV. The authors further identify platypus KHNYN as a potent and broadly active antiviral factor across multiple viruses independent of CpG content.

This study addresses an interesting and important question about why the CG dinucleotide content of avian influenza viruses is not as constrained as that of mammalian adapted influenza viruses and goes on to investigate whether ZAP and KHNYN are drivers of these viral nucleic acid adaptations. While the complementary computational, evolutionary, and structural analyses are novel and compelling, the study is limited by a lack of biochemical and functional validation, including direct evidence of ZAP and KHNYN RNA binding or degradation and mechanistic insights into how platypus KHNYN inhibits avian viruses. As a result, many of the conclusions were not supported by the experimental data and the proposed mechanisms remain speculative dampening the significance of the study.

We appreciate the kind summary and constructive feedback. We agree that our work opens many exciting new directions in this field and hope many others will pursue such important questions along with us. Thanks to the collaborative suggestions provided by the editors, Academic editor, and reviewers we now provide some evidence further supporting our conclusions and providing some mechanistic insight into some of our more novel findings.

Major comments:
1.      The manuscript relies heavily on sophisticated evolutionary and structural analyses to support its conclusions but lacks biochemical validation to directly test its core hypotheses. For example, the authors attribute the inability of avian ZAP to restrict CpG-enriched IAV to divergence in ZAP's second zinc finger (ZF2), yet do not present any RNA binding assays to confirm avian ZAP cannot bind IAV as effectively as mammalian ZAP, nor that ZF2 is the main determinant of this differential binding. Furthermore, even though ZF2 contains the binding pocket for CG dinucleotide, other zinc fingers contain additional binding pockets and both the second and fourth zinc fingers are critical for ZAP's antiviral activity (Guo JVI 2004, Meagher PNAS 2019, Luo Cell Reports 2020). However, this study does not address the potential role of ZF4 and/or other zinc fingers. Similarly, this study proposes KHNYN can restrict CpG-enriched IAV in the absence of ZAP, which contradicts our current understanding of KHNYN as only a ZAP cofactor. This is a novel and interesting finding, however, no evidence is presented that KHNYN can directly bind or degrade RNA independently of ZAP expression. Experiments providing a mechanistic explanation of KHNYN restriction are essential to support this claim. In addition, it is not clear whether the endogenous ZAP protein in the chicken cells is working with overexpressed human KHNYN to inhibit CG-enriched PR8 (Figure 2B). It is also worth noting that the RNA-seq data presented show no reduction in viral mRNA levels in the context of ZAP or KHNYN, despite restriction of IAV. This raises fascinating questions about KHNYN-mediated restriction of IAV. The manuscript discusses KHNYN's mechanism of action through the lens of indirect viral mRNA degradation but does not address the possibility of alternative mechanisms.

We appreciate the comment and now provide evidence that KHNYN may interact with IAV RNA by immunoprecipitation-qPCR (**S6D Fig**) and retains antiviral activity against ROSV even in the absence of chicken ZAP (**S8E Fig**). Furthermore, we were also disappointed that RNA-seq was largely uninformative; however, we now provide evidence that human and platypus KHNYN can inhibit vRNA production observed via IAV minigenome assays (**Fig 4G**). We speculate that these proteins act on vRNA to a greater extent or with greater effect/impact than mRNA (given that vRNA is less abundant); a result which our polyA-enriched RNA-seq libraries would not have shown (IAV vRNA is not polyadenylated). Given the primarily cytoplasmic localization of these proteins, we speculate that IAV vRNA could be targeted during egress (e.g. assembly or packaging) or entry (e.g. vRNA release and cytoplasm-to-nucleus transit). We also show that platypus KHNYN inhibits multiple IAV strains in single cycle or multicycle IAV assays (**Fig 4F** and **S6A-B Figs**). Finally, we have added language in the discussion that expands on speculative KHNYN antiviral mechanisms and highlight future work that might interrogate molecular differences between human and chicken ZAP zinc fingers.

Line 254: “Finally, we observed that human ZAP-S, KHNYN, and dKHNYN may independently interact with PR8CG segment 5 RNA by RNA immunoprecipitation and quantitative PCR (RIP-qPCR) in infected DF-1 cells stably expressing these YFP-tagged proteins (**S6D Fig**).”

Line 351: “We also found similar results for mammalian KHNYN restriction of ROSV in the absence of chicken ZAP using DF-1 cells with ggaZC3HAV1 knocked out by CRISPR/Cas9 (**S8E Fig** and **S4 Fig**).”

Line 463: “Here, we discovered platypus KHNYN as a putative potent and broadly acting antiviral restriction factor. However, it is unclear how mammalian KHNYN functions independently to inhibit IAV or ROSV and how platypus KHNYN functions against such diverse viruses. We show preliminary evidence for human KHNYN interaction with IAV PR8CG Segment 5 RNA, yet platypus KHNYN only weakly enriched for this RNA, albeit at lower protein expression levels. In addition, we observed that platypus KHNYN in particular could inhibit the assembly and release of retroviral virions with typically minimal effects on cellular retroviral Gag expression or loading control proteins. We previously showed that artificial relocalization of HIV-1 viral genomic RNA could inhibit virion assembly and release from cells[87,88]. Our results here might suggest platypus KHNYN may accomplish similar inhibition against many retroviruses.”

Line 447: “Previous work showed chicken ZAP was less specific for CpG dinucleotides within the context of human-chicken chimeric proteins[50]. Future work determining the preferred nucleotide preferences of other ZAP orthologues as well as the amino acid, structural, and domain (zinc finger 1-4) determinants of nucleotide preferences will be critical in understanding host-viral ZAP-RNA interactions and evolution.”

2.      There is an underexplored discrepancy in the isoform-specific restriction of different viruses by ZAP. The authors show that ZAP-S restricts IAV but not Rous sarcoma virus (ROSV), while ZAP-L restricts ROSV but not IAV. This divergence in antiviral activity is fascinating considering ZAP-L is canonically considered the more antiviral isoform of ZAP. The authors should discuss the discrepancy of their data from previous studies especially when both IAV and ROSV are CpG-enriched viruses and both ZAP isoforms contain the RNA binding domain. It would strengthen the manuscript to address isoform-specific restriction of specific viruses, particularly in the context of subcellular localization and cofactor interactions.

We appreciate the comment and agree that this is among our most exciting findings (**Fig 2** [ZAP-S vs IAV] and **Fig 4** [ZAP-L vs ROSV]); which we now discuss much more thoroughly. Our previous discussion devoted half a paragraph to this discrepancy in the field that some viruses are ZAP-S restricted and some are ZAP-L restricted and this is likely dependent on subcellular localization. Importantly, localization is likely the primary driver/determinant of antiviral activity and specificity (particularly Schwerk et al showed ZAP-S +CVIS rescues Sindbis restriction [31740798] and Kmiec et al similarly for CpG-enriched HIV-1 [PMID: 34695163]). Conversely, one can hypothesize (and our data support against IAV) that a ZAP-S (diffuse cytoplasmic) viral restriction would be reduced by limiting ZAP-S to membranes by CVIS. Furthermore, the literature is complicated by mutation, segment, strain, virus, and methodology specific differences that may contribute to the complex and nuanced context of ZAP/KHNYN/CpG associated antiviral activity. We have added more detail to further accentuate isoform-specific and ZAP/KHNYN restriction differences that exist in the literature as well as that subcellular localization of antiviral proteins and targeted viral RNAs must overlap.

Line 451: “Chicken and duck encode primarily prenylated isoforms of ZAP[85], homologous to ZAP-L. We found that only prenylated isoforms of mammalian ZAP and no forms of chicken or duck ZAP restricted ROSV (Fig 4 and S8 Fig). Curiously, the original discovery of ZAP-dependent restriction of MLV identified and used a form of ZAP lacking the carboxy-terminal prenylation motif[86]. We confirmed that mammalian (human, pig, and dog) and avian (chicken and duck) ZAP proteins localized as expected with long or prenylation motif encoding isoforms at endomembranes and short or prenylation mutants exhibiting diffuse cytoplasmic localization (**S9 Fig**). We also note that platypus ZAP is not particularly restrictive against ROSV and has a more diverse CaaX motif, however mutation of C653S appeared to affect its localization. Future work should explore mammalian ZAP restriction of IAV, in particular common host or mixing vessel species (e.g. pig, cow, or dog).”

In addition, we provide additional data related to **S8A Fig** (showing mammalian ZAP-L orthologues restriction of ROSV) in new **S9 Fig**. Importantly, we show the localization of ZAP-L proteins consistent with prenylation and ZAP-S (or CaaX mutant) localization consistent with cytosolic localization using fluorescence microscopy of mNG-tagged ZAP proteins (**S9A Fig**). We also provide the amino acid alignments of the carboxy-terminal prenylation motifs of multiple relevant ZAP-L orthologues (**S9B Fig**). We include further discussion of the limited antiviral activity of platypus ZAP (using prenylated or mutant) against ROSV and also speculate that perhaps this might be balanced/compensated by the enhanced activity of platypus KHNYN.

Line 480: “We might speculate that platypus KHNYN potency might compensate for platypus ZAP impotency observed in our assays against ROSV. Future work examining their relative antiviral activities and interactions will be informative for understanding the evolution of these two restriction factors.”

3.      The finding that platypus KHNYN restricts a broad range of viruses is intriguing and suggests functional divergence from other mammalian KHNYN orthologs. However, the mechanism underlying this potent and broad antiviral activity remains unclear. The authors report that mutation of the NYN catalytic residues does not impair restriction, yet truncation of the endonuclease domain abolishes activity (Figure S7B-C), which is a contradiction that is not fully addressed. Furthermore, although structural modeling reveals a distinct conformation in the KH domain of platypus KHNYN, the functional implications of this difference are not explored. The study would be strengthened by additional experiments to determine whether platypus KHNYN binds viral RNA directly, whether it uses a distinct cofactor, or whether it acts through a different antiviral pathway altogether. These findings are promising but require further mechanistic resolution to clarify how platypus KHNYN achieves its broad antiviral activity.

We appreciate the comment and agree that the platypus KHNYN antiviral activity is quite intriguing! Similar to our response above to Reviewer 2 – Major Comment 1: In the process of providing western blot controls for **Fig 4D** and **S8B Fig** as well as following collaborative suggestions for this revision, we have identified some possible mechanisms underpinning platypus KHNYN activity. We found that platypus KHNYN may inhibit assembly and release of retroviral virions (ROSV, HIV-1, and MLV) from cells in a dose-dependent manner with typically minimal effects on cellular expression of retroviral Gag proteins or loading controls (**Fig 4E** and **S8B-E Fig**). We are intrigued by these results in particular based on our previous work on inhibition of HIV-1 assembly and release (PMID: 28053097; bioRxiv: 846105v2). Also, we found that platypus KHNYN inhibits viral polymerase activity or viral RNA production as suggested by IAV minigenome assay (**Fig 4G**). We note that it’s unclear if or how these activities are related except that many antiviral proteins can exert restriction on viruses through multiple or disparate mechanisms (e.g. APOBEC3G exhibits deaminase dependent and independent restriction of HIV-1 [PMID: 17942420], IFITM3 inhibits IAV entry but MLV envelope incorporation during egress [PMID: 31964738]).

Line 473: “We also found that platypus KHNYN could inhibit PR8CG and OH175 in multi-cycle and/or single-cycle IAV assays. It’s unclear how or if the antiviral mechanisms of platypus KHNYN against IAV versus retroviruses are similar, related, or distinct. However, many antiviral proteins can exert restriction on viruses through multiple or disparate mechanisms. Notably, APOBEC3 proteins can exhibit both deaminase-dependent and deaminase-independent restriction against multiple viruses[89–92]. In addition, IFITM3 inhibits IAV during virus entry but can inhibit MLV during egress and glycoprotein incorporation into progeny virions[93,94].”

We also added language highlighting future directions to understand the implications of the shorter loop above the extended di-KH domain in platypus KHNYN.

Line 483: “In particular, we showed that platypus KHNYN may exhibit a shorter loop above the extended di-KH domain and future work using chimera or mutants might inform greater molecular details and mechanistic implications of platypus KHNYN antiviral activity.”

We agree that the mutational resilience of platypus KHNYN antiviral activity is exciting! We provide an amino acid alignment of the NYN domain for select mammalian KHNYN orthologues showing the conservation of this triad and that the residues selected for mutation were appropriate and expected to inactivate NYN-dependent endonuclease activity (**S10A Fig**). We also provide structural models for human, pig, dog, and platypus NYN domains showing computationally predicted structural similarity (**S10B Fig**).

Line 338: “We observed that the NYN domains of platypus KHNYN, including the aspartic acid catalytic residues, are relatively well conserved by amino acid alignment and computational structural modeling (**S10A-B Fig**).”

Finally, we expanded our discussion/limitation of this curious result and antiviral mechanism for platypus KHNYN as well as highlight that future work by us and others should examine this mechanism including if it uses a distinct cofactor (a cofactor that must be conserved across mammals and avian species cell lines used in our study). In fact, this might be quite exciting that platypus KHNYN could use a cofactor to inhibit viruses that is deeply conserved and expressed in species as divergent as chicken and human cells [across 319 million years of evolution!]. Though, as it stands platypus KHNYN may represent a *bona fide* restriction factor that exhibits cell-autonomous activity. This is not without precedent in that multiple APOBEC3 restriction factors retain activity when expressed across highly divergent cell systems (e.g. PMID: 15823539 showing that human and mouse APOBEC3 proteins can inhibit yeast Ty3 retrotransposons in yeast cells; PMID: 32238588 showing human tetherin inhibits avian leukosis virus when expressed in chicken DF1 cells and chicken tetherin inhibits HIV-1 when expressed in human cells).

Line 486: “Regardless, platypus KHNYN may function as a bona fide cell autonomous restriction factor or potentially use cofactors or cellular features that are conserved across 300 million years of evolution between humans and chickens.”

Line 538: “Many restriction factors exhibit cell-autonomous activity. Human and mouse APOBEC3 proteins function almost universally in vertebrate cell lines against retroviruses as well as in yeast against many Ty3 retrotransposons[89]. Similarly, human TETHERIN restricts avian leukosis viruses when expressed in chicken cells and chicken TETHERIN restricts HIV-1 when expressed in human cells[90].”

4. Figure 1:
a. In panels D, F, G and H, there is a consistent 10-fold decrease in viral PFU/mL between the PR8 and PR8CG experiments across all conditions, including the controls. This implies that there could be some antiviral activity attributed to an unknown or unidentified host factor. This further complicates the claims made by this study, as there might exist another antiviral factor that may interact with KHNYN and/or ZAP. This observation was not discussed in the manuscript.

We appreciate the comment. See above for our response to Reviewer 1, Minor Comment 3. In addition, we highlight in our discussion/limitations that future work by us and others should examine what host cofactors across many vertebrate species influence the antiviral activity of different ZAP and/or KHNYN orthologues.

Line 543: “Future work should examine what cofactors, proteins, and/or RNA molecules interact[100] with mammalian and avian ZAP as well as mammalian KHNYN across different cellular contexts and their impact on antiviral activity.”

5. Figure 2:
a. The colors used for figures in most panels are difficult to distinguish clearly. For example, the color used for "TRIM25" and "gTRIM25" in panel A are very similar and thus was briefly confusing. Moreover, the shades of green used for ZAP-L and ZAP-S blend together and this is made more confusing by the slightly different shades of these colors used for the mutant ZAPs in panels B, D and F.

We appreciate the comment and have adjusted our color/pattern scheme throughout the figures for clarity.

6. Figure S2:
a. The analyses in Figure S2 present data that appear to contradict the manuscript's emphasis on CpG as a uniquely targeted and selectively depleted dinucleotide in mammalian-adapted IAV. Several other dinucleotides, including UpC and GpG, exhibit similar variability across IAV infecting different hosts as CpG, raising questions about whether CpG content is uniquely constrained. The manuscript would benefit from additional discussion on broader dinucleotide usage trends and whether CpG-specific selection pressure is unique.

We appreciate the comment and note that CpG is of interest in this study while others have previously explored different mono-, di-, tri-, or poly-nucleotide signatures differentially present in viruses, bacteria, and eukaryotic pathogens (PMID: 32797103). We now provide greater discussion of this topic and the evolutionary/adaptive context more broadly. Specifically, we believe that while much of our knowledge on host-pathogen interactions has focused on Red-Queen conflicts highlighted by protein-protein interactions and dN/dS-based metrics of positive selection – future work should explore other molecular interactions as surfaces for evolutionary conflict (e.g. RNA-protein and RNA-RNA).

Line 414: “Furthermore, we focused on CpG dinucleotides yet other dinucleotide, trinucleotide, or polynucleotide motifs under- or over-represented in host-virus relationships should be examined systematically [72–75]. In addition, much of our knowledge on host-pathogen interactions has focused on Red Queen conflicts highlighted by protein-protein interactions and non-synonymous/synonymous codon (dN/dS) based metrics of positive selection[9,76–79]. Future work should explore other molecular interactions as surfaces for evolutionary conflict (e.g. RNA-protein and RNA-RNA)[80–82].”

------------------------------------

Reviewer #3:

This study by Becker et al examines the roles of ZAP and KHNYN in restricting viruses with RNA genomes high in CpG content. They show the mammalian versions of these proteins act independently as restriction factors against an engineered version of the PR8 strain of IAV that has elevated CpG content in one of the gene segments. They do not observe this effect with avian origin homologs. Based on the well documented observation that avian origin viruses tend to have higher CpG content and viruses that circulate in humans appear to be under selection to decrease CpG content, the authors argue that the high CpG content-dependence of ZAP/KHNYN-mediated restriction of PR8 reveals a general restriction factor that may act to protect mammals from avian origin viruses. They go on to show that the avian retrovirus ROSV is also sensitive to restriction by mammalian ZAP and KHNYN. Finally, they also clearly show that the restriction activities of ZAP and KHNYN are independent of each other.

The significance of the study comes from the in-depth molecular analysis of ZAP and KHNYN function and specificity and the clarification of their independent functions. The experiments are well designed and controlled, and the data generally support the conclusions of the paper.  My only concern is that the differences in restriction between lab adapted PR8 and PR8cg are used to make fairly sweeping, general conclusions about species barriers. The language should be toned down at points and the limitations section fleshed out to reflect this.

We appreciate the kind summary and supportive feedback. We have mollified our language throughout or provided more narrow/specific language when possible. We also tested an avian IAV strain (OH175) using the chicken DF1 cells expressing human ZAP-S and KHNYN in **S6A-B Figs**. We believe this strikes an appropriate balance in highlighting our novel findings using PR8 and PR8_CG_ extensively while providing more data with an avian relevant virus strains.

Examples include:

Line 374: “We showed that chicken ZAP as well as duck ZAP may not restrict IAV, in particular PR8 or PR8_CG_ (**Fig 1** and **S3-4 Figs**)”

Line 194: “**Human ZAP-S and KHNYN can independently restrict CpG-enriched PR8 IAV.”**

Line 214: “Thus, we reasoned that avian species may be defective and/or deficient in two restriction factor genes: ZAP as well as KHNYN.”

Line 253: “Human ZAP-S and KHNYN also modestly restricted an avian IAV strain (OH175) with a modest but natural CpG-enriched PA segment (**S6A-C Fig**).”

Line 522: “Throughout this study we primarily use a common laboratory strain of IAV and a previously published CpG-enriched segment 5 mutant strain (PR8 and PR8CG) as well as in some assays an avian strain (OH175) and human pandemic strain (Cal04). Future work will benefit from examining ZAP, KHNYN, and TRIM25 susceptibility of many influenza strains derived from humans, wild birds, agricultural poultry, and domestic mammals as well as emerging mammalian H5N1 IAV isolates[96].”

Minor points:
Text on fig 2A hard to read

We appreciate this comment and a related comment from Reviewer 1. We have removed this subpanel.

Fig 3C: any explanation for decreased replication in ZAP2 cells?

We appreciate this question and agree it is curious. This may be due to clonal effects – these CRISPR KO’s are single cell clones and ZAP2 is somewhat divergent in replication activity. This underlies why we tested multiple clones for each gene KO target as well as supports why pooled KO is sometimes more informative. Notably, the same guide was used to generate the ZAP KO in the double knockout cells (ZK1 and ZK2) that allowed for increased replication across two independent clones. We provide this context in the manuscript.

Line 294: “We tested two single cell clones per gene targeted for knockout to assess potential clonal effects and we hypothesize that the decreased replication in ZAP KO 2 may be a clonal effect. The same guide target (ZAP guide 2) was used to make the double knockouts that allowed for increased replication across two independent clones.”

Line 356: PR8 is not a human IAV

We appreciate the lack of clarity in this statement and revised our language. That line referred to CpG content of human IAV sequences rather than the particular laboratory-adapted PR8 strain used throughout the paper.

Line 373: “Here, we observed that human IAV sequences exhibit a marked decrease in CpG content relative to avian IAV sequences (**Fig 1A** and **S1-2 Figs**).”
