# Supplementary material for: Mammalian antiviral proteins ZAP and KHNYN can independently restrict CpG-enriched avian viruses
Source: PLoS Biol. 2025 Oct 28;23(10):e3003471. doi: 10.1371/journal.pbio.3003471 (PMC12582503; doi:10.1371/journal.pbio.3003471)
Supplement: Attachment — Submitted filename: Becker ZKT Response Rev2 PBIO V1.docx [file pbio.3003471.s022.docx]

------------------------------------------------------------------------
EDITOR RESPONSE
------------------------------------------------------------------------

Dear Jordan,

Thank you for your patience while we considered your revised manuscript "Mammalian ZAP and KHNYN can independently restrict CpG-enriched avian viruses" for publication as a Research Article at PLOS Biology. This revised version of your manuscript has been evaluated by the PLOS Biology editors, the Academic Editor and the original reviewers.

Based on the reviews, we are likely to accept this manuscript for publication, provided you satisfactorily address the remaining editorial requests. Please also make sure to address the following data and other policy-related requests.

a) We routinely suggest changes to titles to ensure maximum accessibility for a broad, non-specialist readership, and to ensure they reflect the contents of the paper. In this case, we would suggest a minor edit to the title, as follows. Please ensure you change both the manuscript file and the online submission system, as they need to match for final acceptance:
"Mammalian antiviral proteins ZAP and KHNYN can independently restrict CpG-enriched avian viruses"

Thank you for the suggestion and appreciate the new title’s increased audience accessibility.

b) Please add the link of the funding agencies in the Financial Disclosure statement in the manuscript details.

We will provide this during submission:

NIAID: <https://www.niaid.nih.gov/>

CEIRR: <https://www.ceirr-network.org/>

c) You may be aware of the PLOS Data Policy, which requires that all data be made available without restriction: <http://journals.plos.org/plosbiology/s/data-availability>. For more information, please also see this editorial: <http://dx.doi.org/10.1371/journal.pbio.1001797>
Please supply the numerical values either in the a supplementary file or as a permanent DOI’d deposition for the following figures:
Figure 1ACDFGH, 2A-D, 3CDE, 4ACDEFG, S1A-D, S2A-D, S3EFG, S4C-I, S7BC, S8A-E (and S6ACD)
NOTE: the numerical data provided should include all replicates AND the way in which the plotted mean and errors were derived (it should not present only the mean/average values).

We have provided these values in S2 Table (experimental data) or indicated that they can be found in indicated repository (bioinformatic data with R code for generating figure subpanels; <https://doi.org/10.5281/zenodo.14919910>). In addition, we added language describing how means and standard deviations were calculated in our methods (Statistical Analysis subheading).

d) Please cite the location of the data clearly in all relevant main and supplementary Figure legends, e.g. “The data underlying this Figure can be found in S1 Data” or “The data underlying this Figure can be found in <https://doi.org/10.5281/zenodo.XXXXX>”

We have added this language as requested throughout.

e) Please ensure that you are using best practice for statistical reporting and data presentation. These are our guidelines <https://journals.plos.org/plosbiology/s/best-practices-in-research-reporting#loc-statistical-reporting>  and a useful resource  on data presentation <https://journals.plos.org/plosbiology/article?id=10.1371/journal.pbio.1002128>

-- If you are reporting experiments where n ≤ 5, please plot each individual data point.

We have updated figures to include individual data points.

f) We require the original, uncropped and minimally adjusted images supporting all blot and gel results reported in the Figures 2E-H, 3B, 4CDE, S3CD, S8A-E (and S6BD)

-- We will require these files before a manuscript can be accepted so please prepare and upload them now. Please carefully read our guidelines for how to prepare and upload this data: <https://journals.plos.org/plosbiology/s/figures#loc-blot-and-gel-reporting-requirements>

We have provided these images in S1_raw_images.pdf and included this as a Supplementary Information file.

\#####

As you address these items, please take this last chance to review your reference list to ensure that it is complete and correct. If you have cited papers that have been retracted, please include the rationale for doing so in the manuscript text, or remove these references and replace them with relevant current references. Any changes to the reference list should be mentioned in the cover letter that accompanies your revised manuscript.

In addition to these revisions, you may need to complete some formatting changes, which you will receive in a follow up email. A member of our team will be in touch with a set of requests shortly. If you do not receive a separate email within a few days, please assume that checks have been completed, and no additional changes are required.

We expect to receive your revised manuscript within two weeks.

To submit your revision, please go to <https://www.editorialmanager.com/pbiology/> and log in as an Author. Click the link labelled 'Submissions Needing Revision' to find your submission record. Your revised submission must include the following:
-  a cover letter that should detail your responses to any editorial requests, if applicable, and whether changes have been made to the reference list
-  a Response to Reviewers file that provides a detailed response to the reviewers' comments (if applicable, if not applicable please do not delete your existing 'Response to Reviewers' file.)
-  a track-changes file indicating any changes that you have made to the manuscript.

NOTE: If Supporting Information files are included with your article, note that these are not copyedited and will be published as they are submitted. Please ensure that these files are legible and of high quality (at least 300 dpi) in an easily accessible file format. For this reason, please be aware that any references listed in an SI file will not be indexed. For more information, see our Supporting Information guidelines:
<https://journals.plos.org/plosbiology/s/supporting-information> 

*Published Peer Review History*
Please note that you may have the opportunity to make the peer review history publicly available. The record will include editor decision letters (with reviews) and your responses to reviewer comments. If eligible, we will contact you to opt in or out. Please see here for more details:
<https://plos.org/published-peer-review-history/>

*Press*
Should you, your institution's press office or the journal office choose to press release your paper, please ensure you have opted out of Early Article Posting on the submission form. We ask that you notify us as soon as possible if you or your institution is planning to press release the article.

*Protocols deposition*
To enhance the reproducibility of your results, we recommend that if applicable you deposit your laboratory protocols in [protocols.io](http://protocols.io/), where a protocol can be assigned its own identifier (DOI) such that it can be cited independently in the future. Additionally, PLOS ONE offers an option for publishing peer-reviewed Lab Protocol articles, which describe protocols hosted on [protocols.io](http://protocols.io/). Read more information on sharing protocols at <https://plos.org/protocols?utm_medium=editorial-email&utm_source=authorletters&utm_campaign=protocols>


Please do not hesitate to contact me should you have any questions.

Sincerely,

Melissa

Melissa Vazquez Hernandez, Ph.D.
Associate Editor
[mvazquezhernandez@plos.org](mailto:mvazquezhernandez@plos.org)
PLOS Biology

------------------------------------------------------------------------
REVIEWERS' COMMENTS
------------------------------------------------------------------------


Reviewer #1: The authors have addressed adequately the previous concerns

Reviewer #2: The authors have thoroughly and thoughtfully addressed all my prior concerns.


Reviewer #3: I feel the concerns raised by all three reviewers have been adequately addressed

To all editors and reviewers, thank you again for your time, effort, and appreciation of our work.
